# Supplementary material for: A 2-hydroxybutyrate-mediated feedback loop regulates muscular fatigue
Source: eLife. 2024 Sep 3;12:RP92707. doi: 10.7554/eLife.92707 (PMC11371357; doi:10.7554/eLife.92707)

Figure 4C – C2C12 confirm overexpression of DDK-tagged hBCAT2

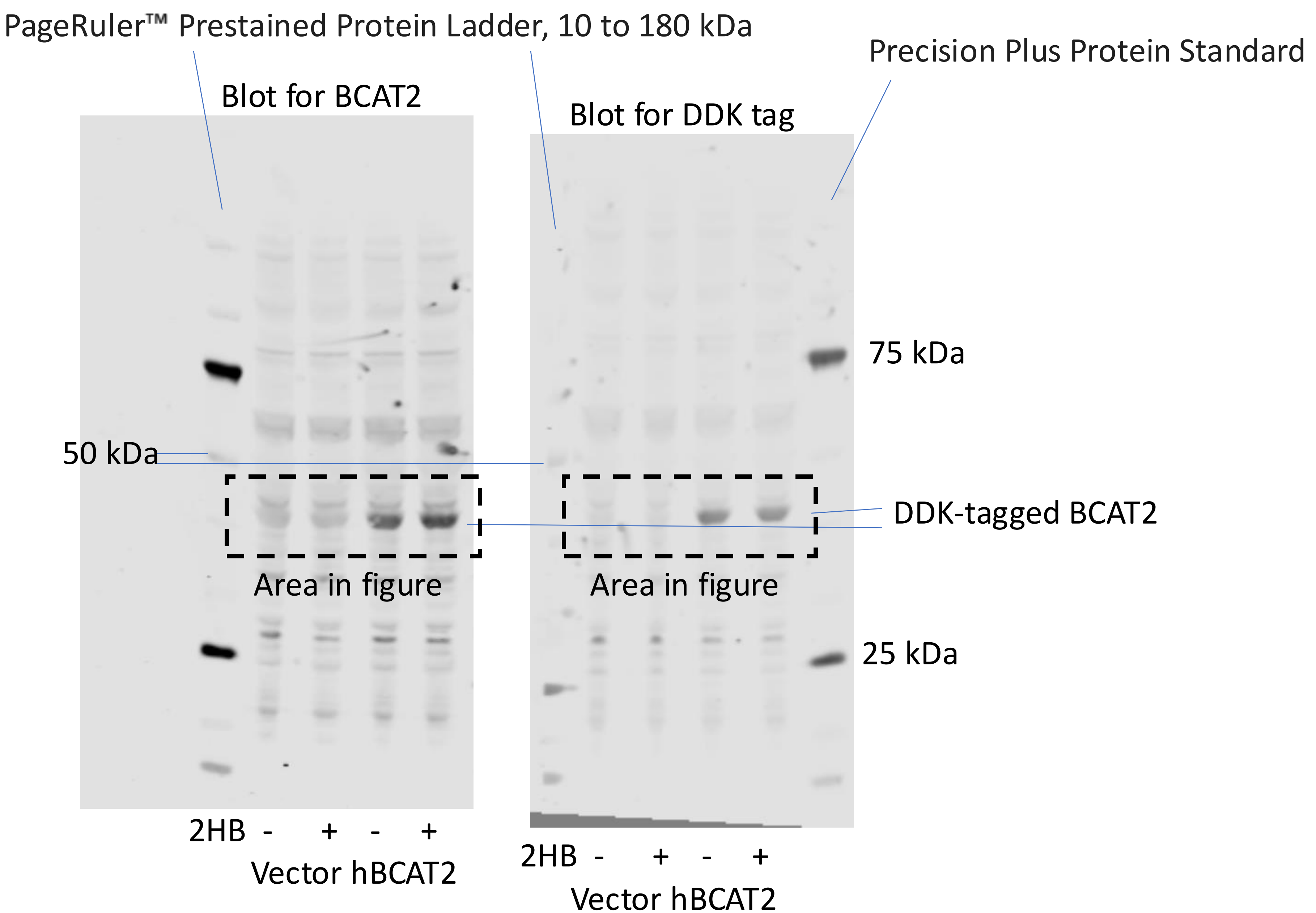

Figure 4E – C2C12 lysates transfected with siRNA against SIRT4 for non-targeting control, stained for total ADPr

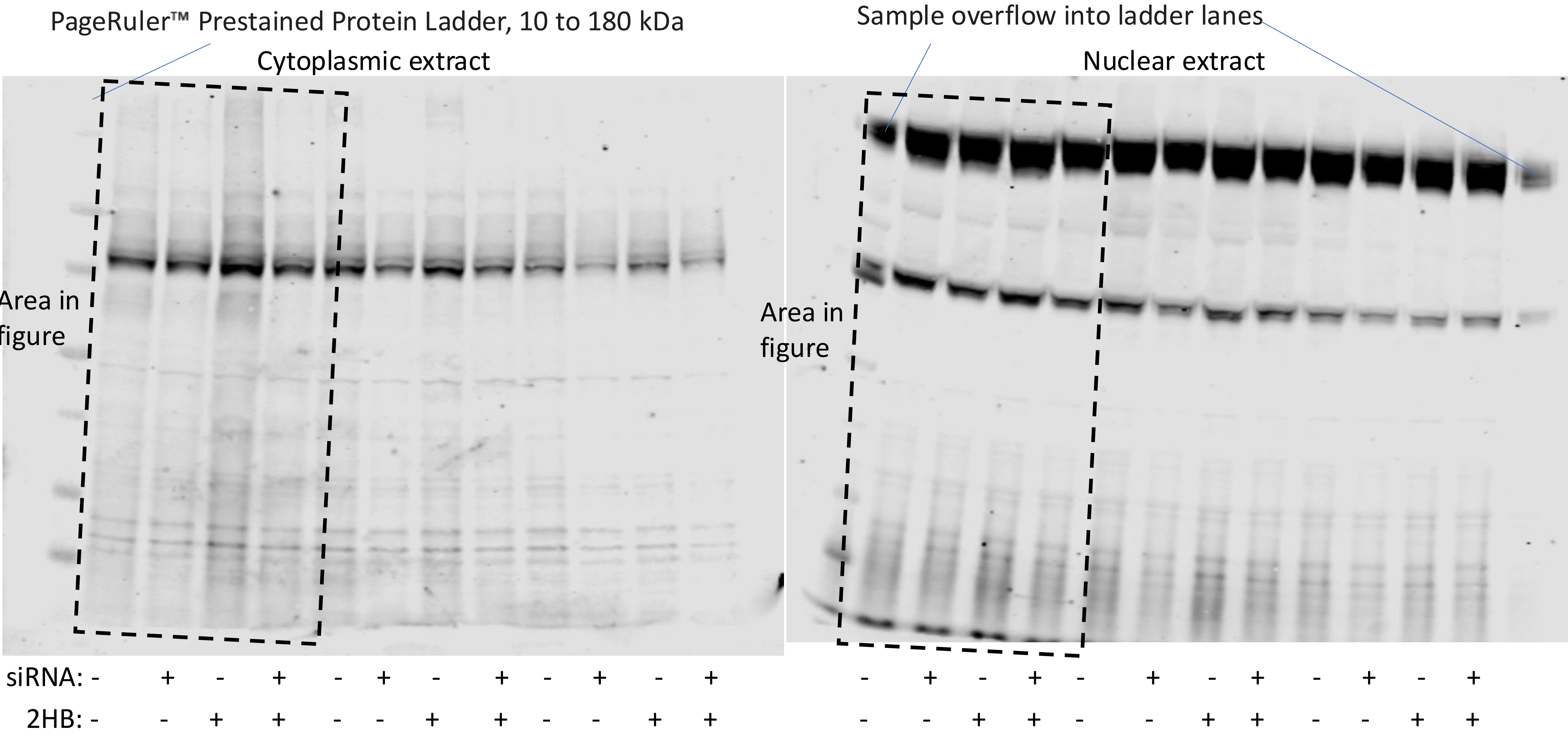

Figure 4F – C2C12 lysates transfected with siRNA against SIRT4 for non-targeting control, IP experiments

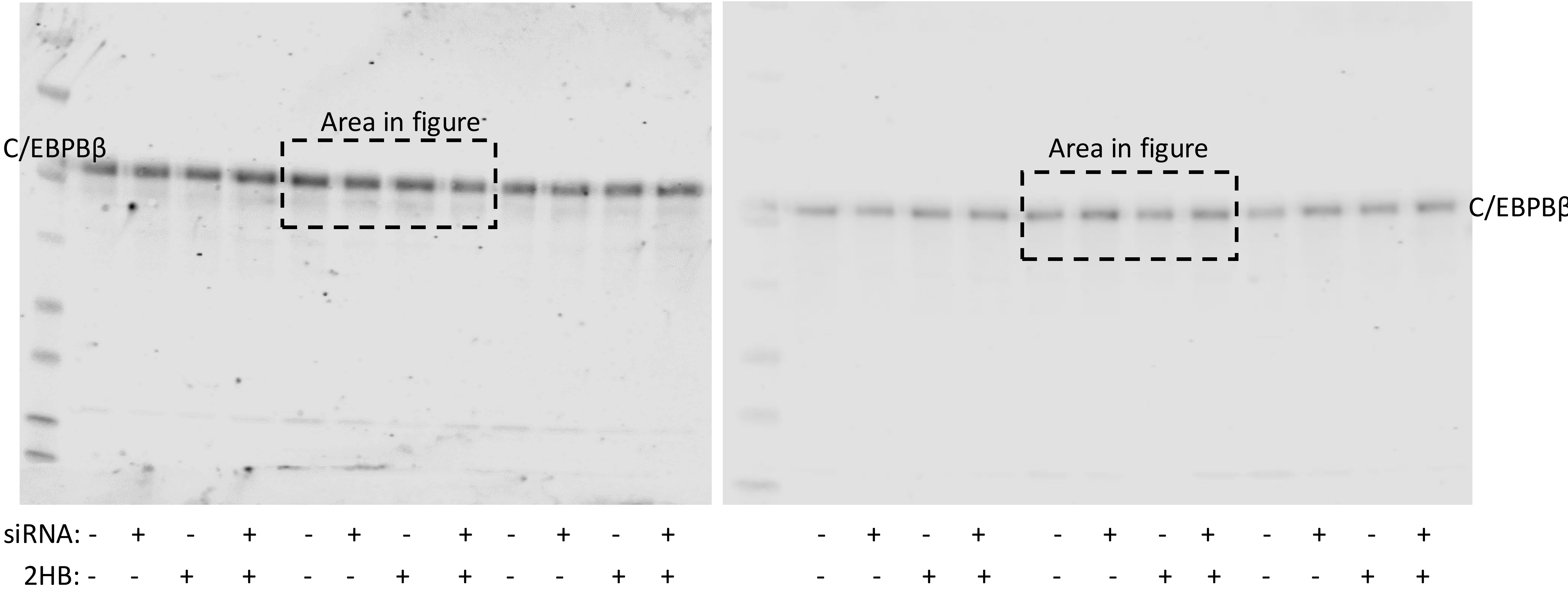

Figure 4F continued – C2C12 lysates transfected with siRNA against SIRT4 for non-targeting control, IP experiments, input samples blot for C/EBPbeta and H3

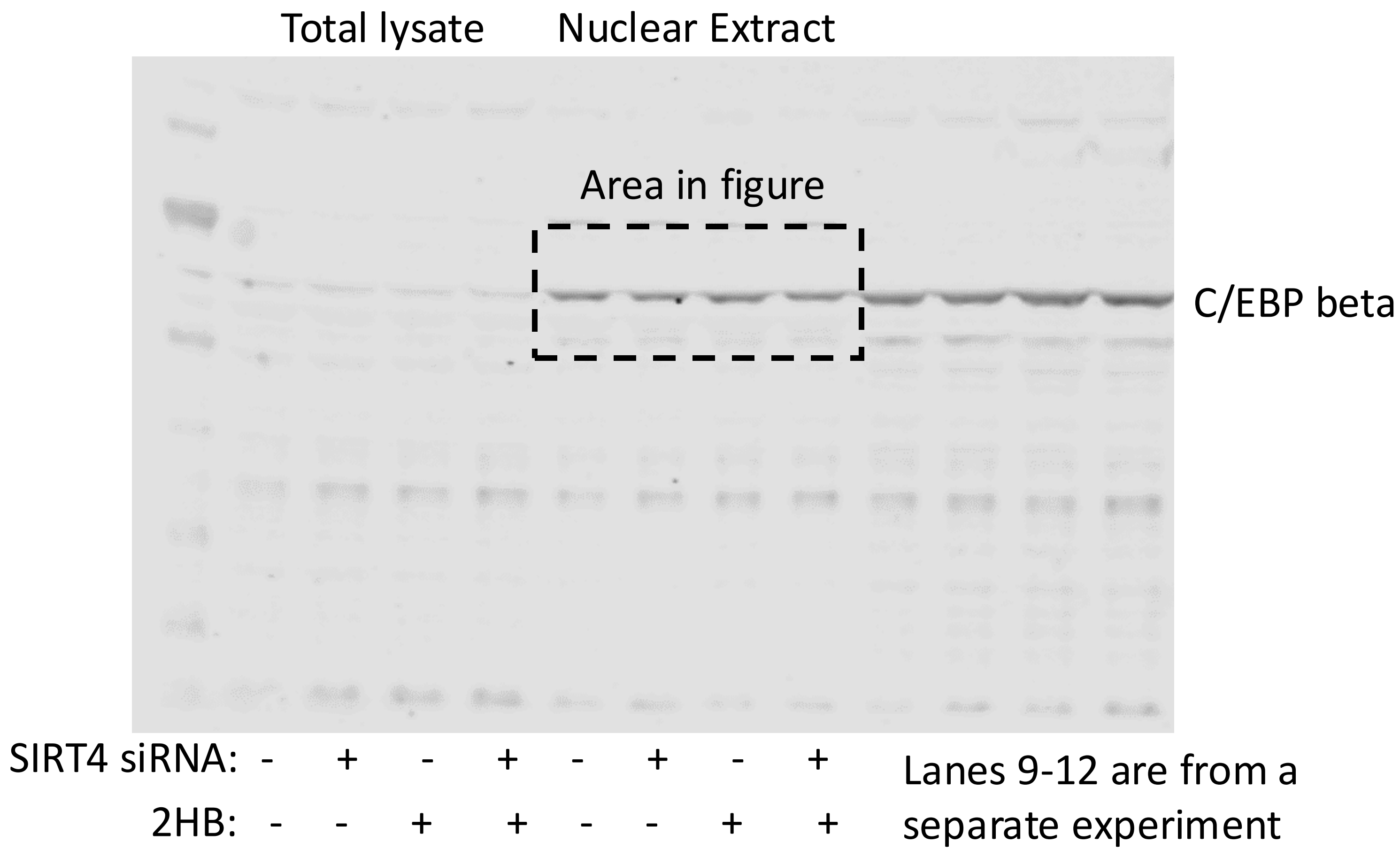

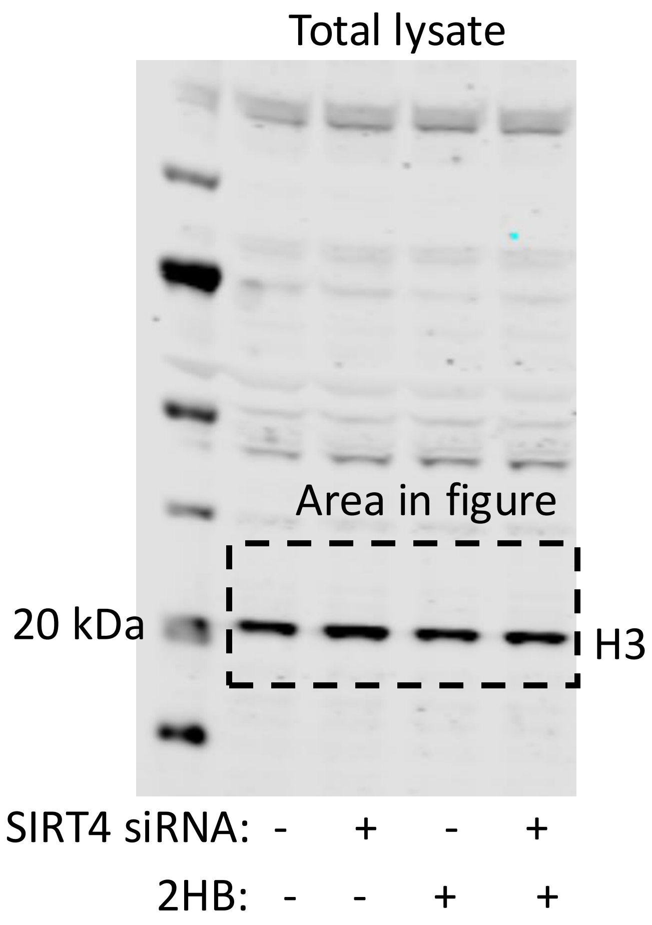

Supplement: Figure 4—source data 2. [file elife-92707-fig4-data2.zip › ANNOTATED WESTERN BLOTS.pdf]
