# Supplementary figures and images for: A 2-hydroxybutyrate-mediated feedback loop regulates muscular fatigue
Source: eLife. 2024 Sep 3;12:RP92707. doi: 10.7554/eLife.92707 (PMC11371357; doi:10.7554/eLife.92707)

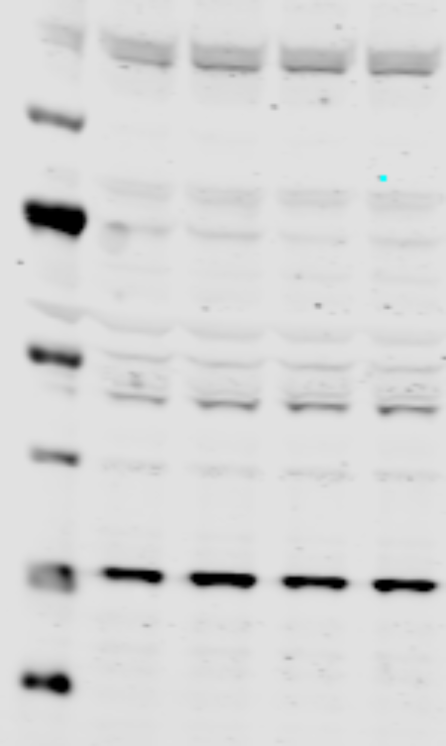

Supplement: Figure 4—source data 3. [file elife-92707-fig4-data3.zip › Figure 4 - Source Data - Unlabelled Western Blots/FIG4F_4.png]

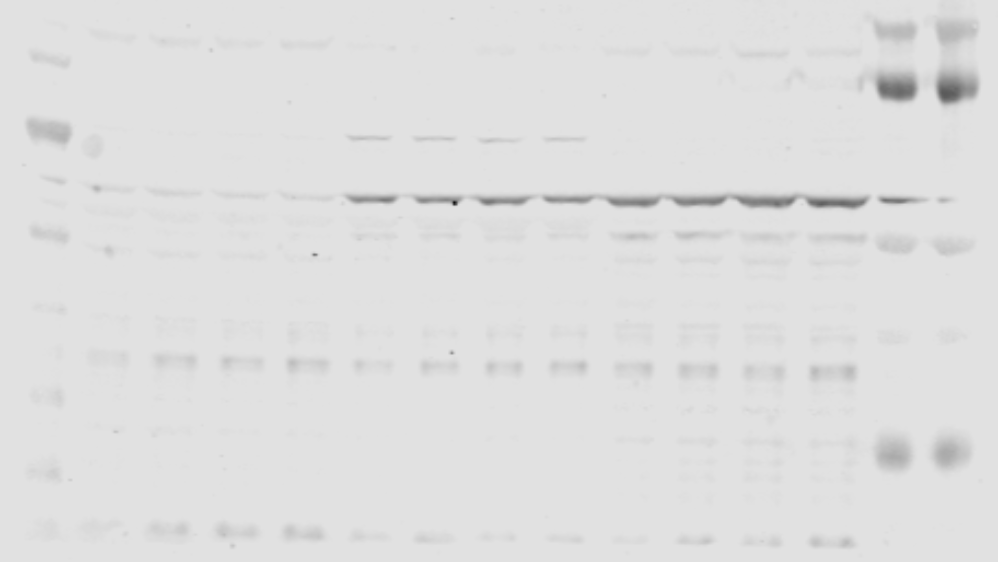

Supplement: Figure 4—source data 3. [file elife-92707-fig4-data3.zip › Figure 4 - Source Data - Unlabelled Western Blots/FIG4F_3.png]

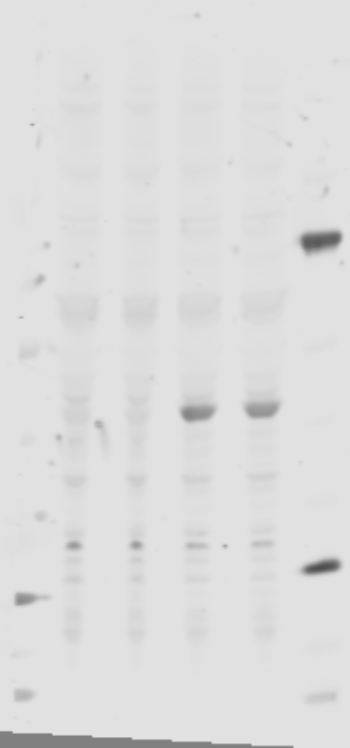

Supplement: Figure 4—source data 3. [file elife-92707-fig4-data3.zip › Figure 4 - Source Data - Unlabelled Western Blots/FIG4C.png]

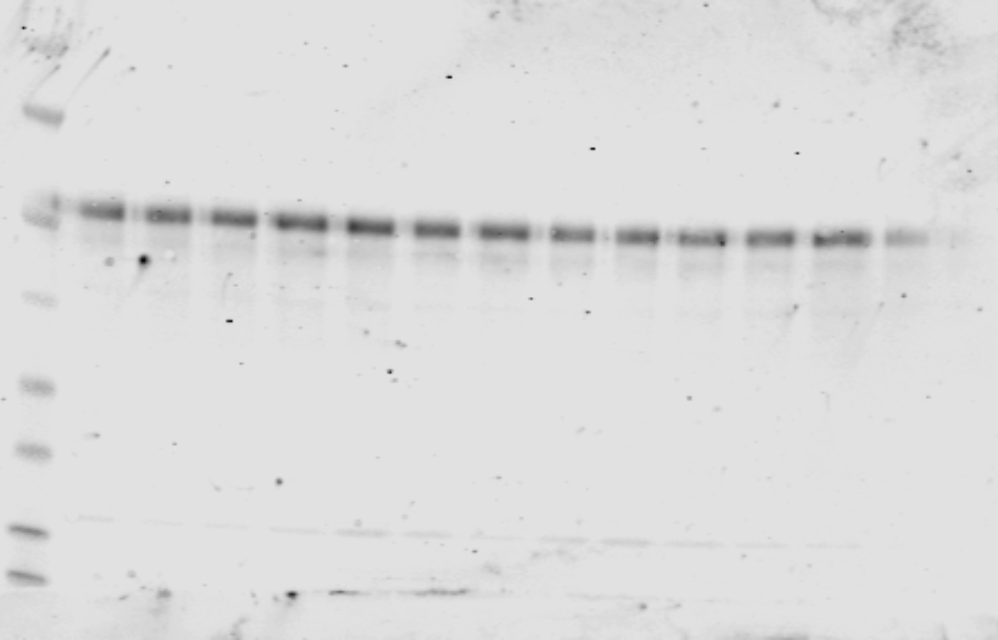

Supplement: Figure 4—source data 3. [file elife-92707-fig4-data3.zip › Figure 4 - Source Data - Unlabelled Western Blots/FIG4F_2.png]

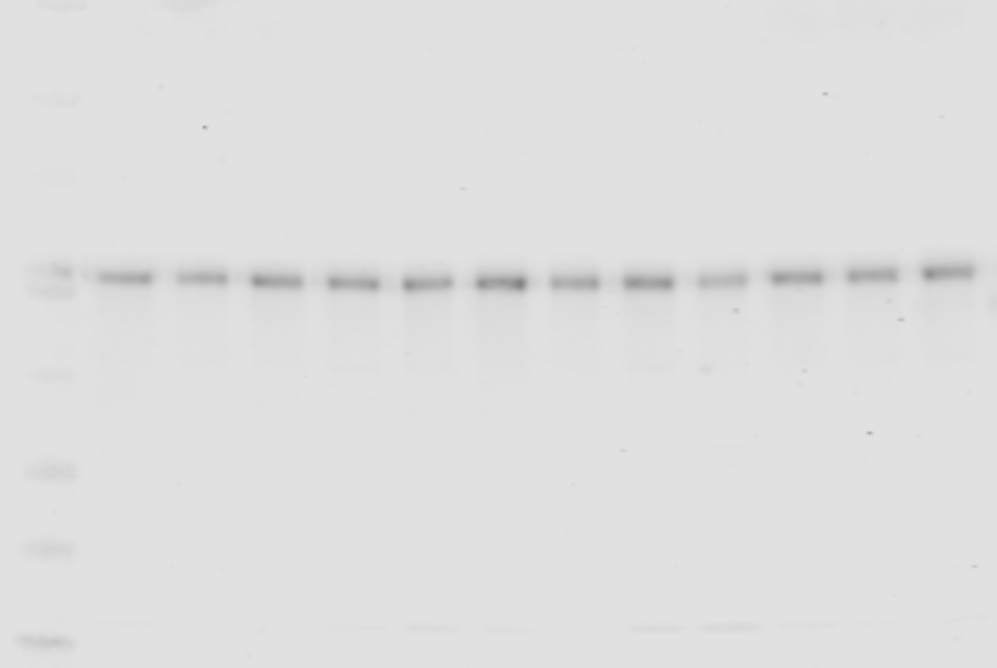

Supplement: Figure 4—source data 3. [file elife-92707-fig4-data3.zip › Figure 4 - Source Data - Unlabelled Western Blots/FIG4F_1.png]

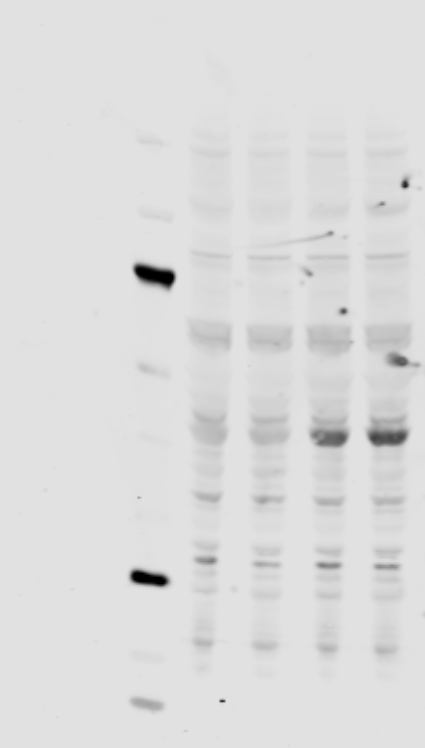

Supplement: Figure 4—source data 3. [file elife-92707-fig4-data3.zip › Figure 4 - Source Data - Unlabelled Western Blots/FIG4C_2.png]

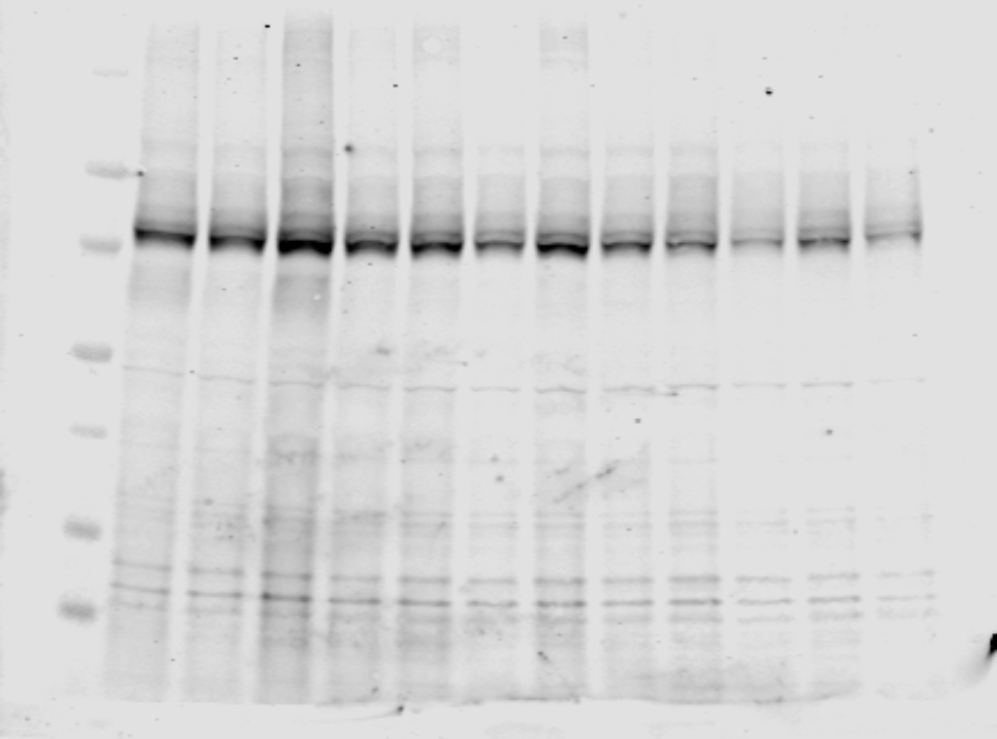

Supplement: Figure 4—source data 3. [file elife-92707-fig4-data3.zip › Figure 4 - Source Data - Unlabelled Western Blots/FIG4E_2.png]

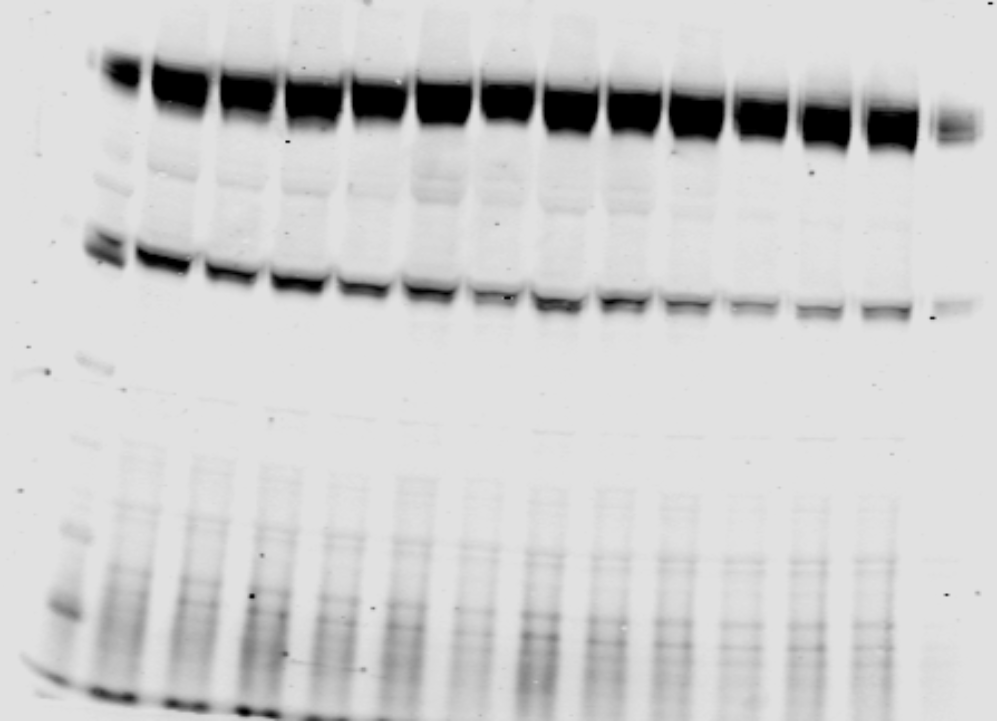

Supplement: Figure 4—source data 3. [file elife-92707-fig4-data3.zip › Figure 4 - Source Data - Unlabelled Western Blots/FIG4E_1.png]

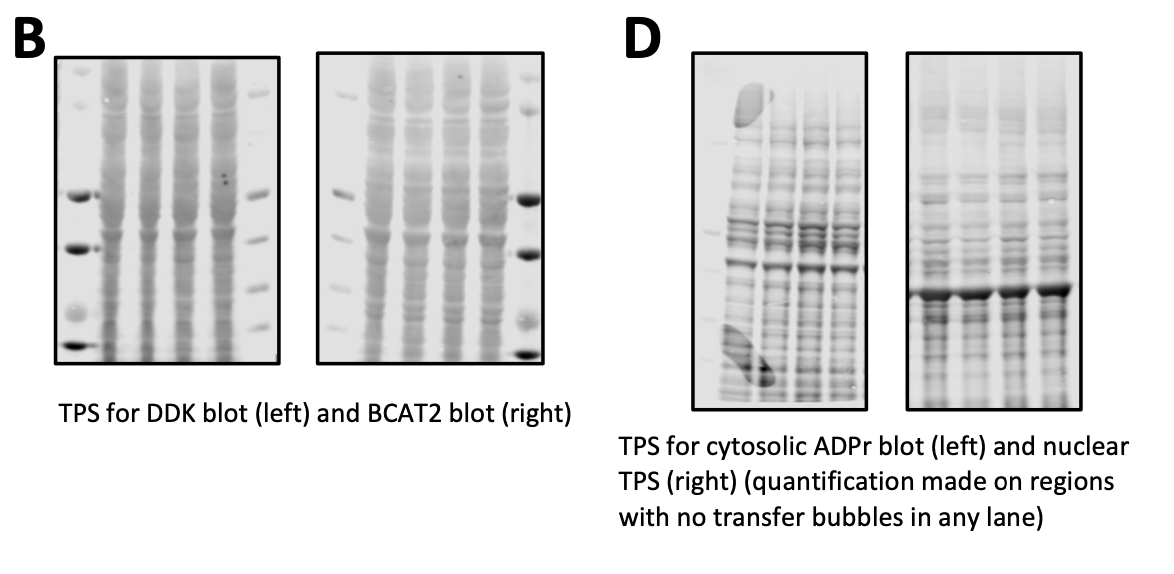

Supplement: Figure 4—figure supplement 1—source data 2. [file elife-92707-fig4-figsupp1-data2.zip › Annotated Western Blots/Figure 4 - figure supplement 1 - western blot annotations.png]

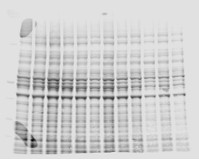

Supplement: Figure 4—figure supplement 1—source data 3. [file elife-92707-fig4-figsupp1-data3.zip › Western Blots/Figure4_supplement1_D_left_TPS.jpg]

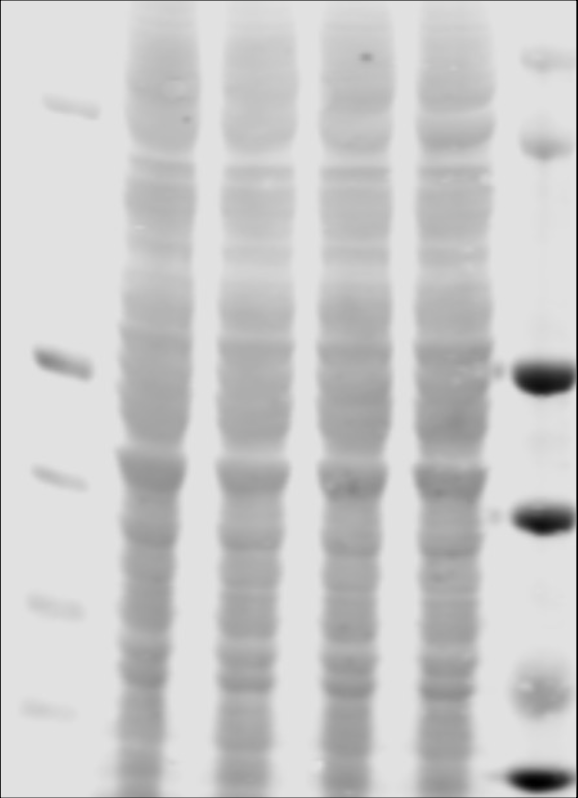

Supplement: Figure 4—figure supplement 1—source data 3. [file elife-92707-fig4-figsupp1-data3.zip › Western Blots/Figure 4_Supplement_1_TPS_right.png]

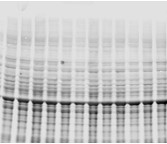

Supplement: Figure 4—figure supplement 1—source data 3. [file elife-92707-fig4-figsupp1-data3.zip › Western Blots/Figure4_supplement1_D_right_TPS.jpg]

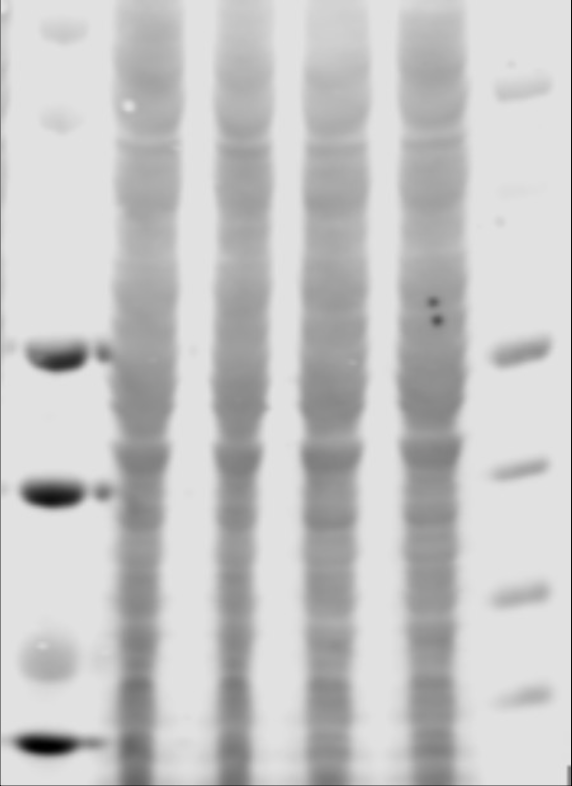

Supplement: Figure 4—figure supplement 1—source data 3. [file elife-92707-fig4-figsupp1-data3.zip › Western Blots/Figure 4_Supplement_1_TPS_left.png]

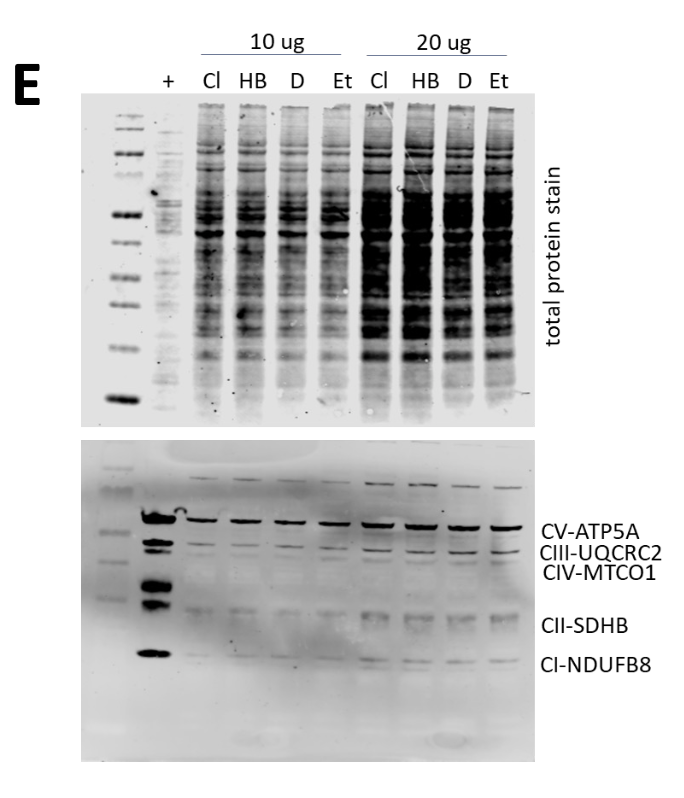

Supplement: Figure 5—figure supplement 1—source data 2. [file elife-92707-fig5-figsupp1-data2.zip › Figure 5 - figure supplement 1 - Source Data - Labelled Western Blots/Figure 5 - supplement 1 - E left.png]

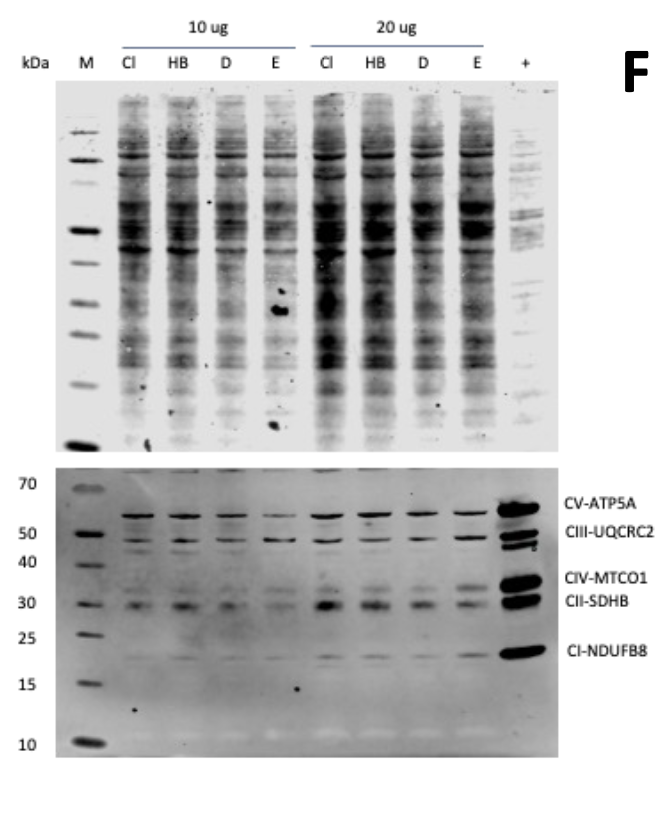

Supplement: Figure 5—figure supplement 1—source data 2. [file elife-92707-fig5-figsupp1-data2.zip › Figure 5 - figure supplement 1 - Source Data - Labelled Western Blots/Figure 5 - supplement 1 - E right.png]

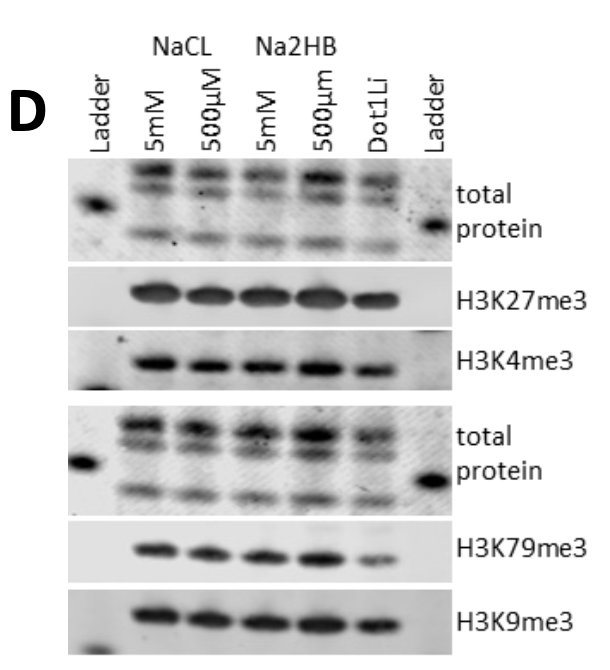

Supplement: Figure 5—figure supplement 1—source data 2. [file elife-92707-fig5-figsupp1-data2.zip › Figure 5 - figure supplement 1 - Source Data - Labelled Western Blots/Figure 5 - supplement 1 - D.png]

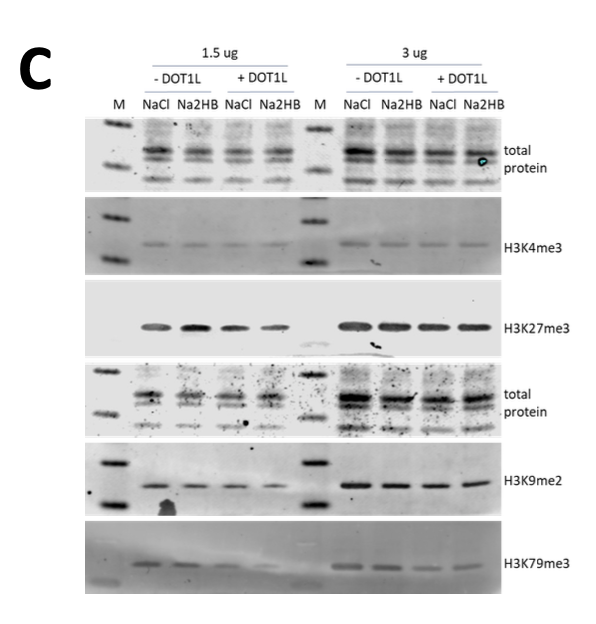

Supplement: Figure 5—figure supplement 1—source data 2. [file elife-92707-fig5-figsupp1-data2.zip › Figure 5 - figure supplement 1 - Source Data - Labelled Western Blots/Figure 5 - supplement 1 - C.png]

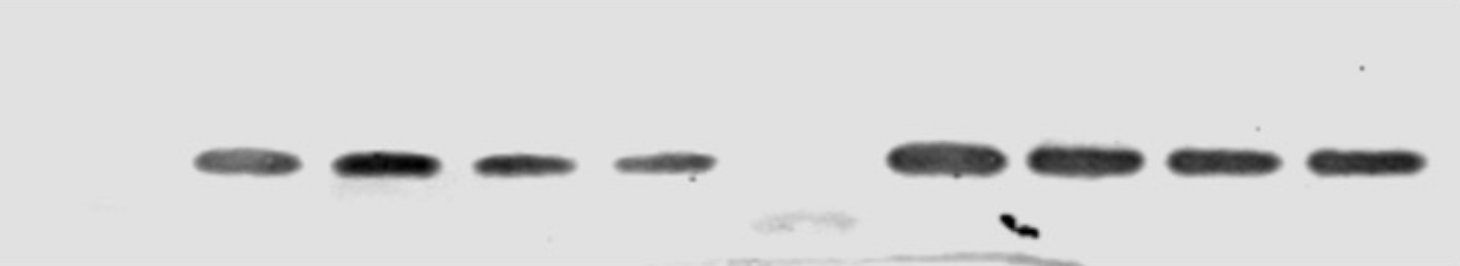

Supplement: Figure 5—figure supplement 1—source data 3. [file elife-92707-fig5-figsupp1-data3.zip › Figure 5 - figure supplement 1 - Source Data - Unlabelled Western Blots/Figure 5 - supplement 1_C_H3K37.png]

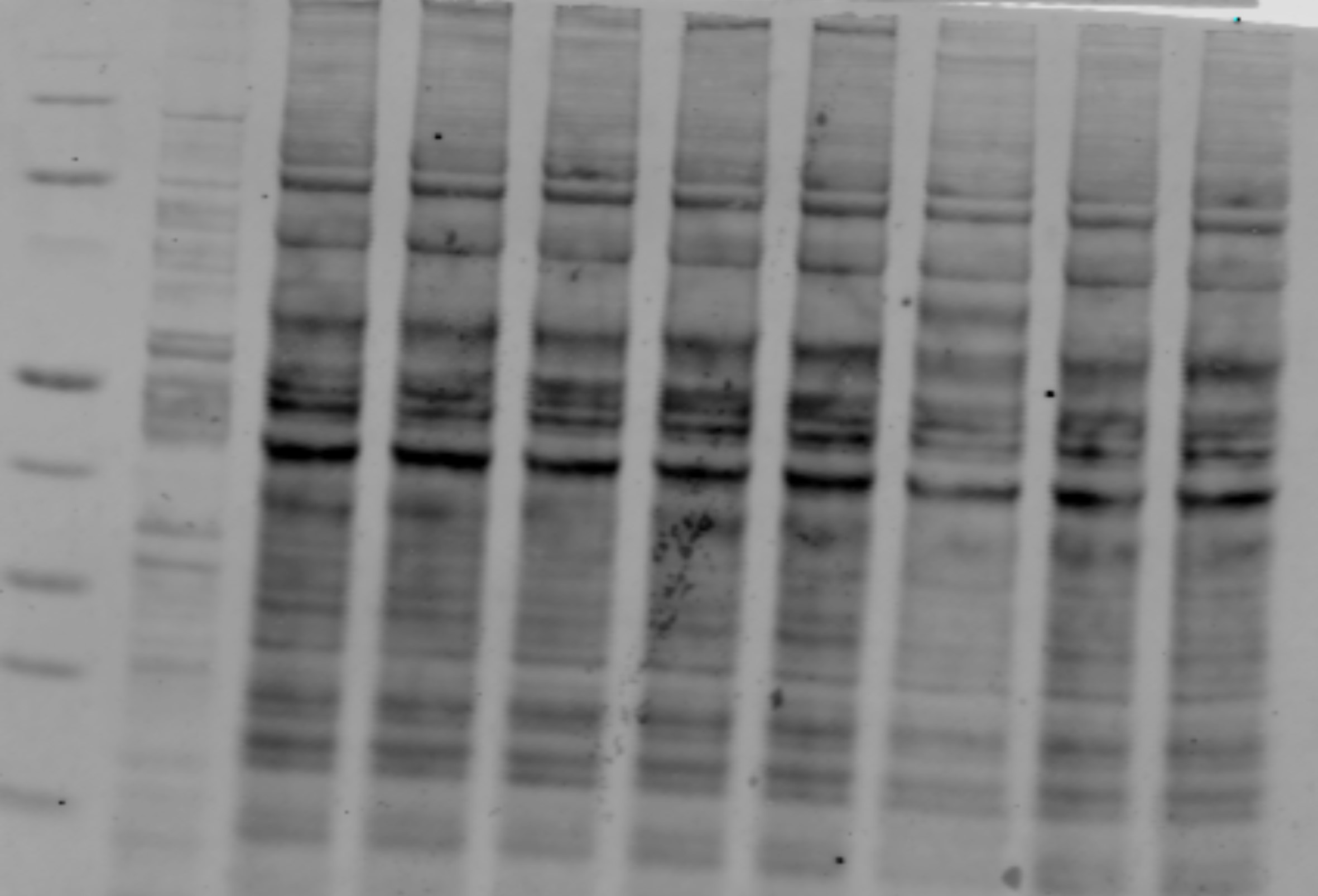

Supplement: Figure 5—figure supplement 1—source data 3. [file elife-92707-fig5-figsupp1-data3.zip › Figure 5 - figure supplement 1 - Source Data - Unlabelled Western Blots/Figure 5_supplement1_E_left_TPS.jpg]

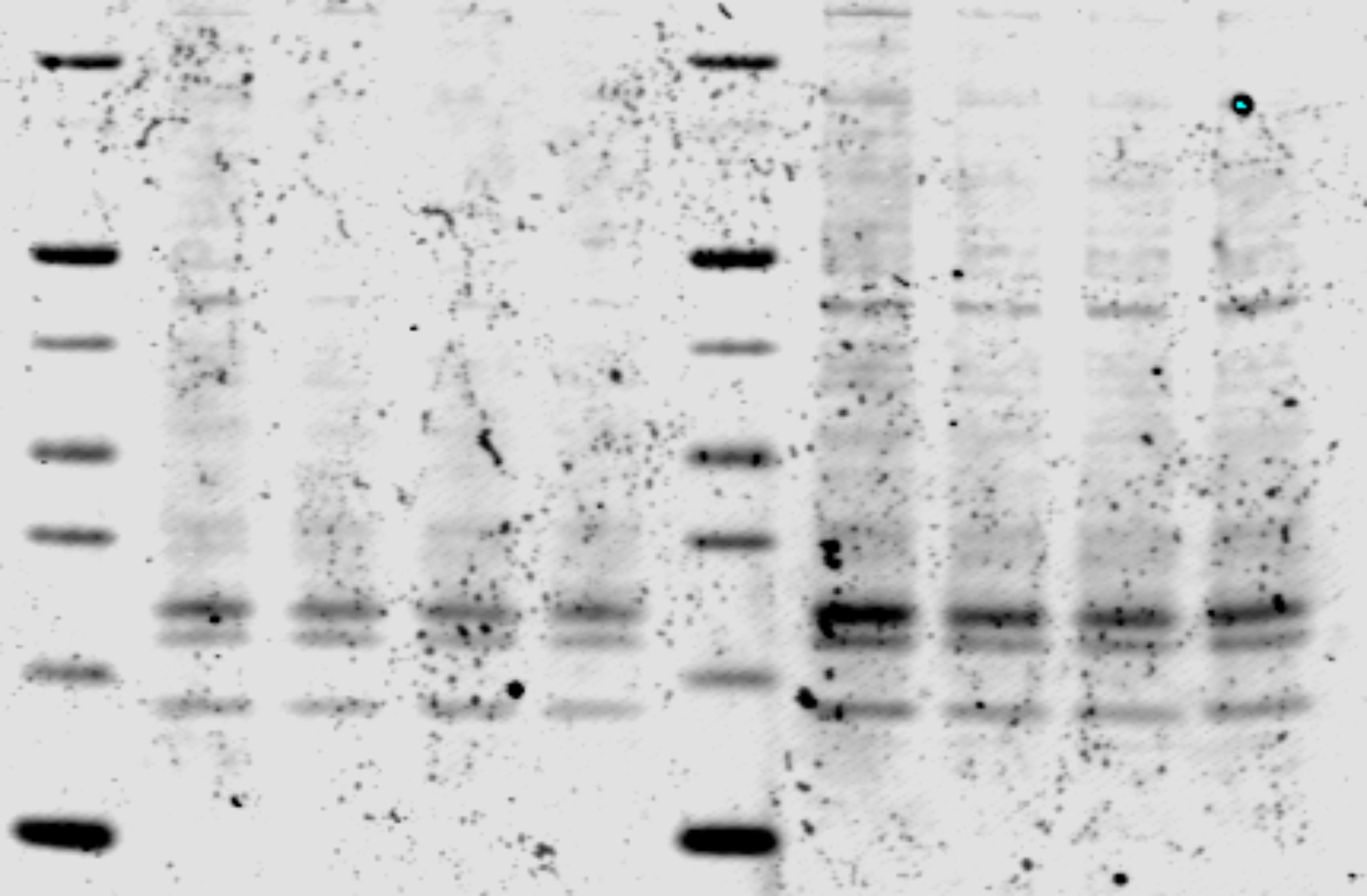

Supplement: Figure 5—figure supplement 1—source data 3. [file elife-92707-fig5-figsupp1-data3.zip › Figure 5 - figure supplement 1 - Source Data - Unlabelled Western Blots/Figure 5_supplement1_C_bottom_TPS.jpg.jpg]

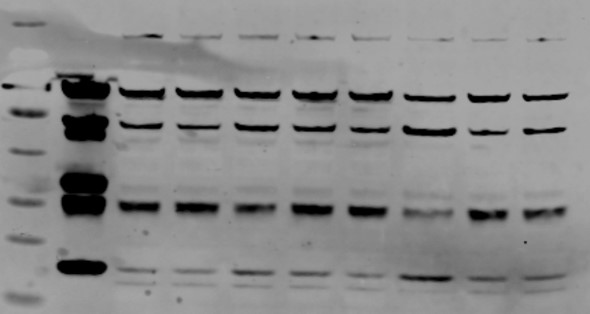

Supplement: Figure 5—figure supplement 1—source data 3. [file elife-92707-fig5-figsupp1-data3.zip › Figure 5 - figure supplement 1 - Source Data - Unlabelled Western Blots/Figure 5_supplement1_E_left_OXPHOS.jpg]

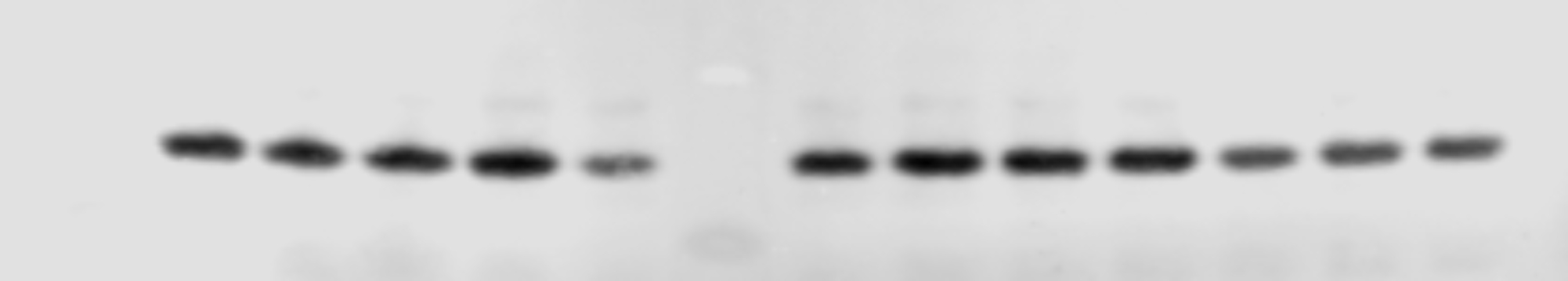

Supplement: Figure 5—figure supplement 1—source data 3. [file elife-92707-fig5-figsupp1-data3.zip › Figure 5 - figure supplement 1 - Source Data - Unlabelled Western Blots/Figure 5_supplement1_D_H3K79me3_H3K9me2.jpg.jpg]

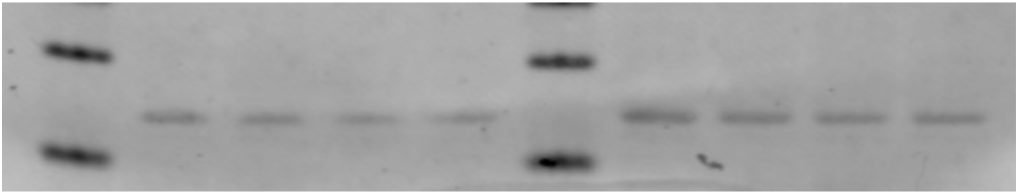

Supplement: Figure 5—figure supplement 1—source data 3. [file elife-92707-fig5-figsupp1-data3.zip › Figure 5 - figure supplement 1 - Source Data - Unlabelled Western Blots/Figure5_supplement1_C_H3K4me3.png]

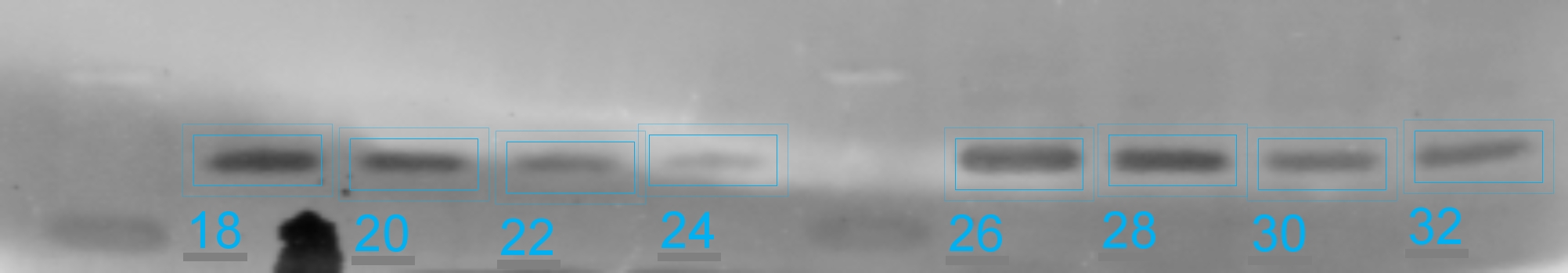

Supplement: Figure 5—figure supplement 1—source data 3. [file elife-92707-fig5-figsupp1-data3.zip › Figure 5 - figure supplement 1 - Source Data - Unlabelled Western Blots/Figure 5_supplement1_C_bottom_H3K9me2.jpg.jpg]

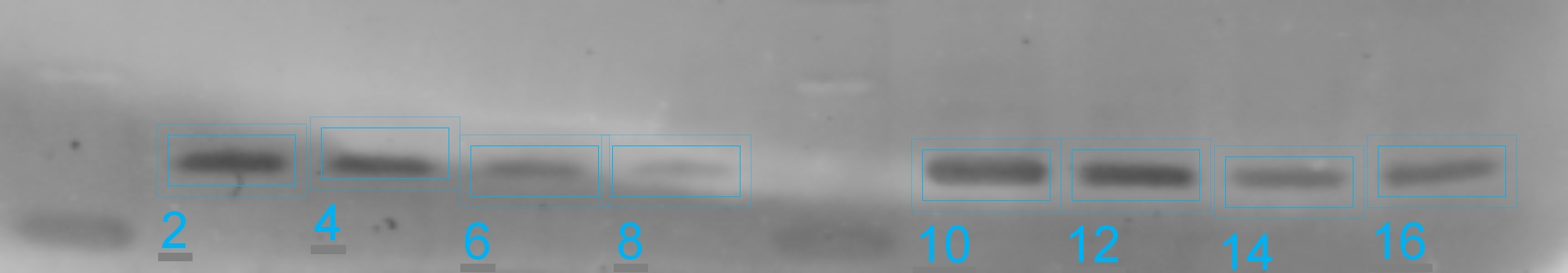

Supplement: Figure 5—figure supplement 1—source data 3. [file elife-92707-fig5-figsupp1-data3.zip › Figure 5 - figure supplement 1 - Source Data - Unlabelled Western Blots/Figure 5_supplement1_C_bottom_H3K79me3.jpg.jpg]

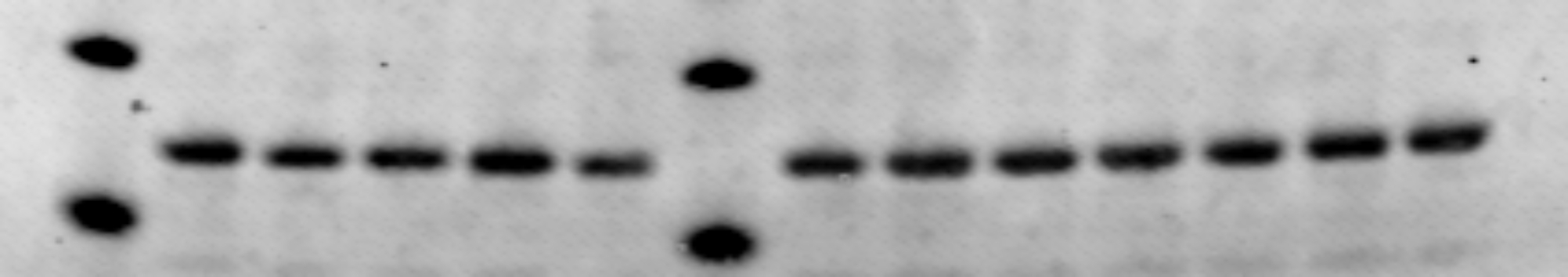

Supplement: Figure 5—figure supplement 1—source data 3. [file elife-92707-fig5-figsupp1-data3.zip › Figure 5 - figure supplement 1 - Source Data - Unlabelled Western Blots/Figure 5_supplement1_D_H3K27me3_H3K4me3.jpg.jpg]

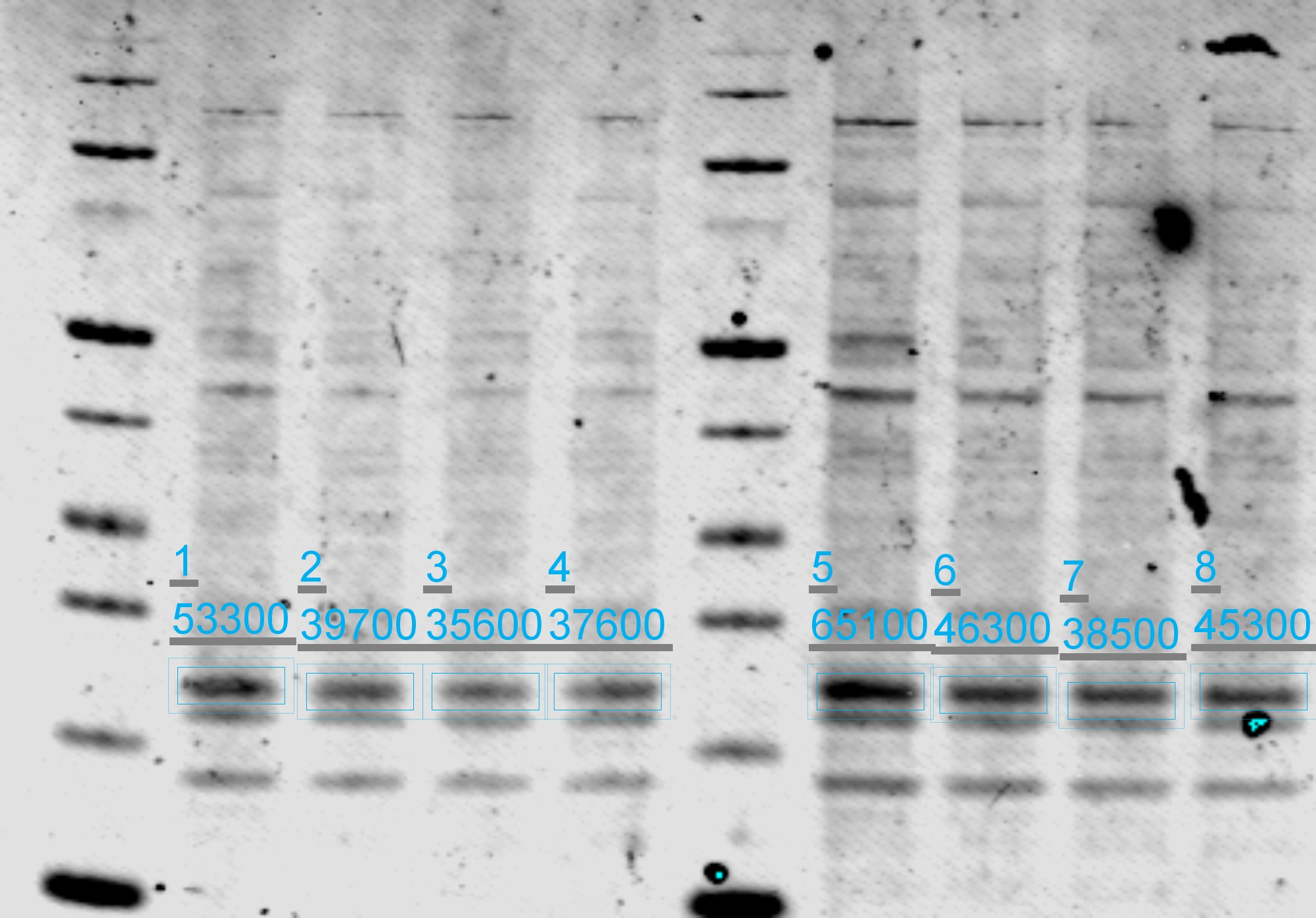

Supplement: Figure 5—figure supplement 1—source data 3. [file elife-92707-fig5-figsupp1-data3.zip › Figure 5 - figure supplement 1 - Source Data - Unlabelled Western Blots/Figure 5_supplement1_C_TPS.jpg.jpg]

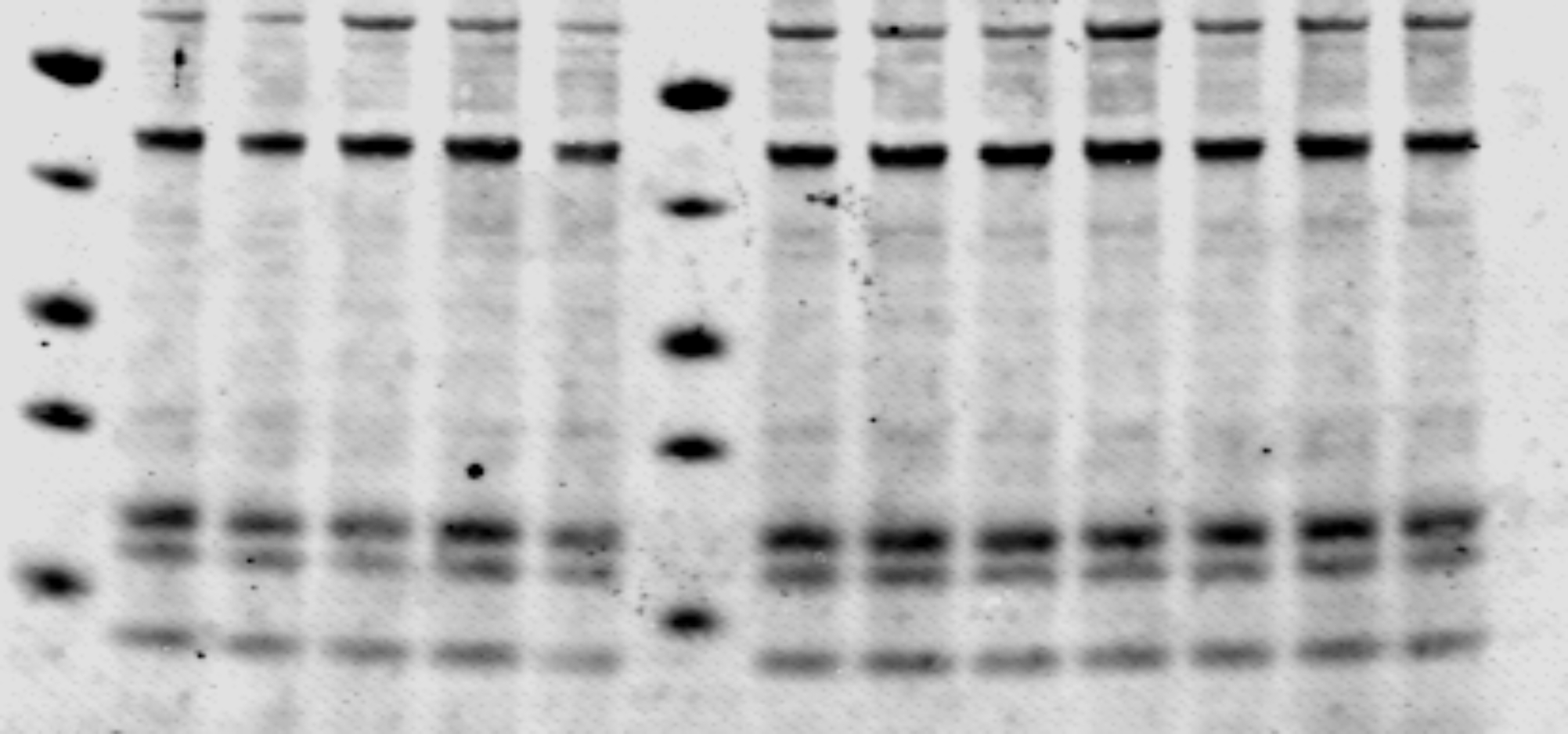

Supplement: Figure 5—figure supplement 1—source data 3. [file elife-92707-fig5-figsupp1-data3.zip › Figure 5 - figure supplement 1 - Source Data - Unlabelled Western Blots/Figure 5_supplement1_D_TPS.jpg.jpg]

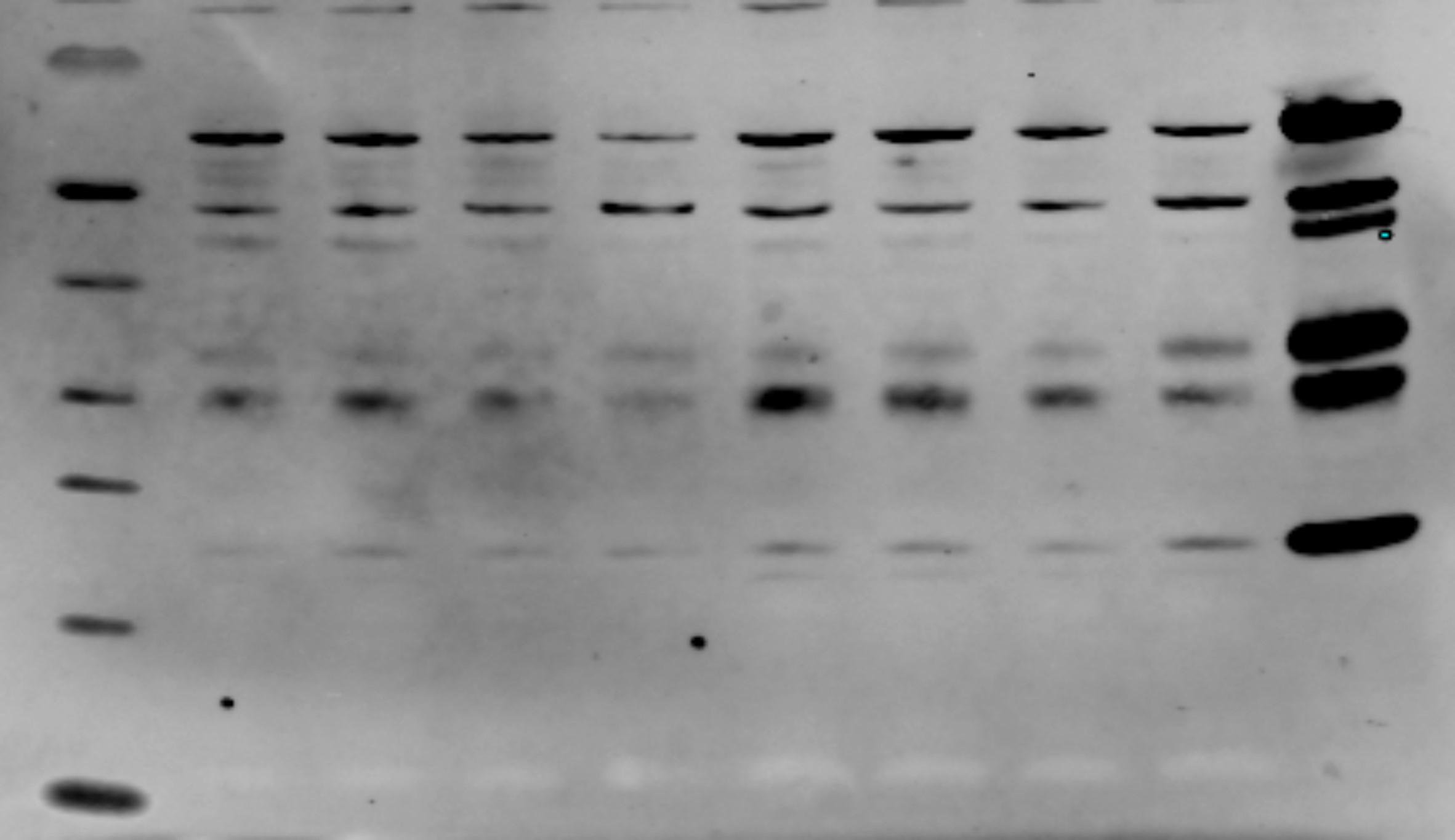

Supplement: Figure 5—figure supplement 1—source data 3. [file elife-92707-fig5-figsupp1-data3.zip › Figure 5 - figure supplement 1 - Source Data - Unlabelled Western Blots/Figure 5_supplement1_E_right_OXPHOS.jpg]

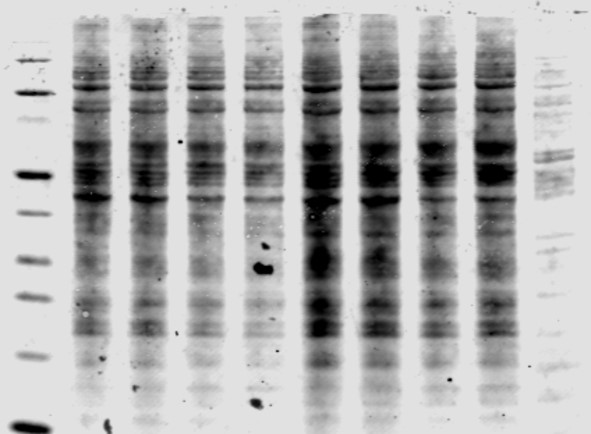

Supplement: Figure 5—figure supplement 1—source data 3. [file elife-92707-fig5-figsupp1-data3.zip › Figure 5 - figure supplement 1 - Source Data - Unlabelled Western Blots/Figure 5_supplement1_E_right_TPS.jpg]

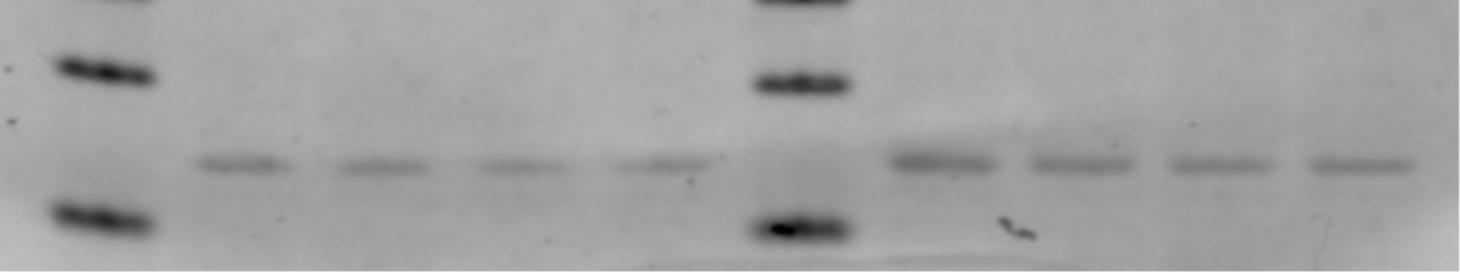

Supplement: Figure 5—figure supplement 1—source data 3. [file elife-92707-fig5-figsupp1-data3.zip › Figure 5 - figure supplement 1 - Source Data - Unlabelled Western Blots/Figure 5 - supplement 1_C_H3K4.png]

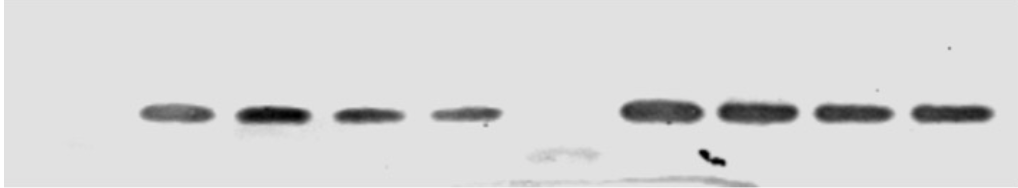

Supplement: Figure 5—figure supplement 1—source data 3. [file elife-92707-fig5-figsupp1-data3.zip › Figure 5 - figure supplement 1 - Source Data - Unlabelled Western Blots/Figure5_supplement1_C_H3K27me3.png]

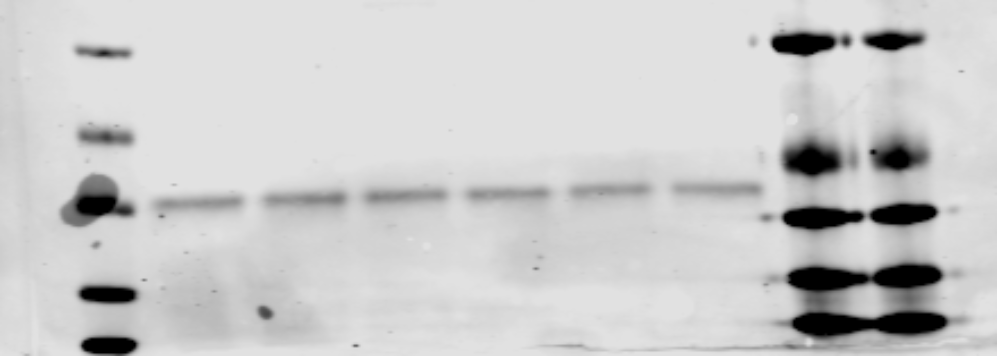

Supplement: Figure 6—source data 3. [file elife-92707-fig6-data3.zip › Figure 6 - Source Data - Unlabelled Western Blots/FIG6B_4.png]

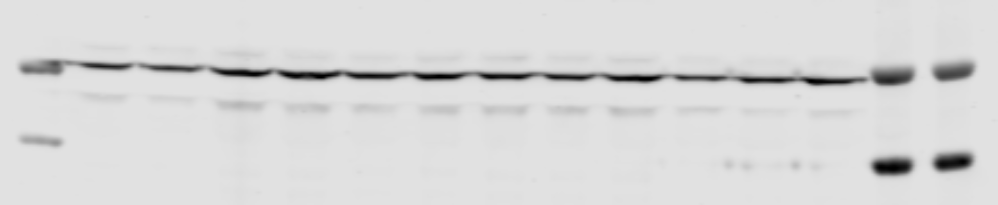

Supplement: Figure 6—source data 3. [file elife-92707-fig6-data3.zip › Figure 6 - Source Data - Unlabelled Western Blots/FIG6D_2.png]

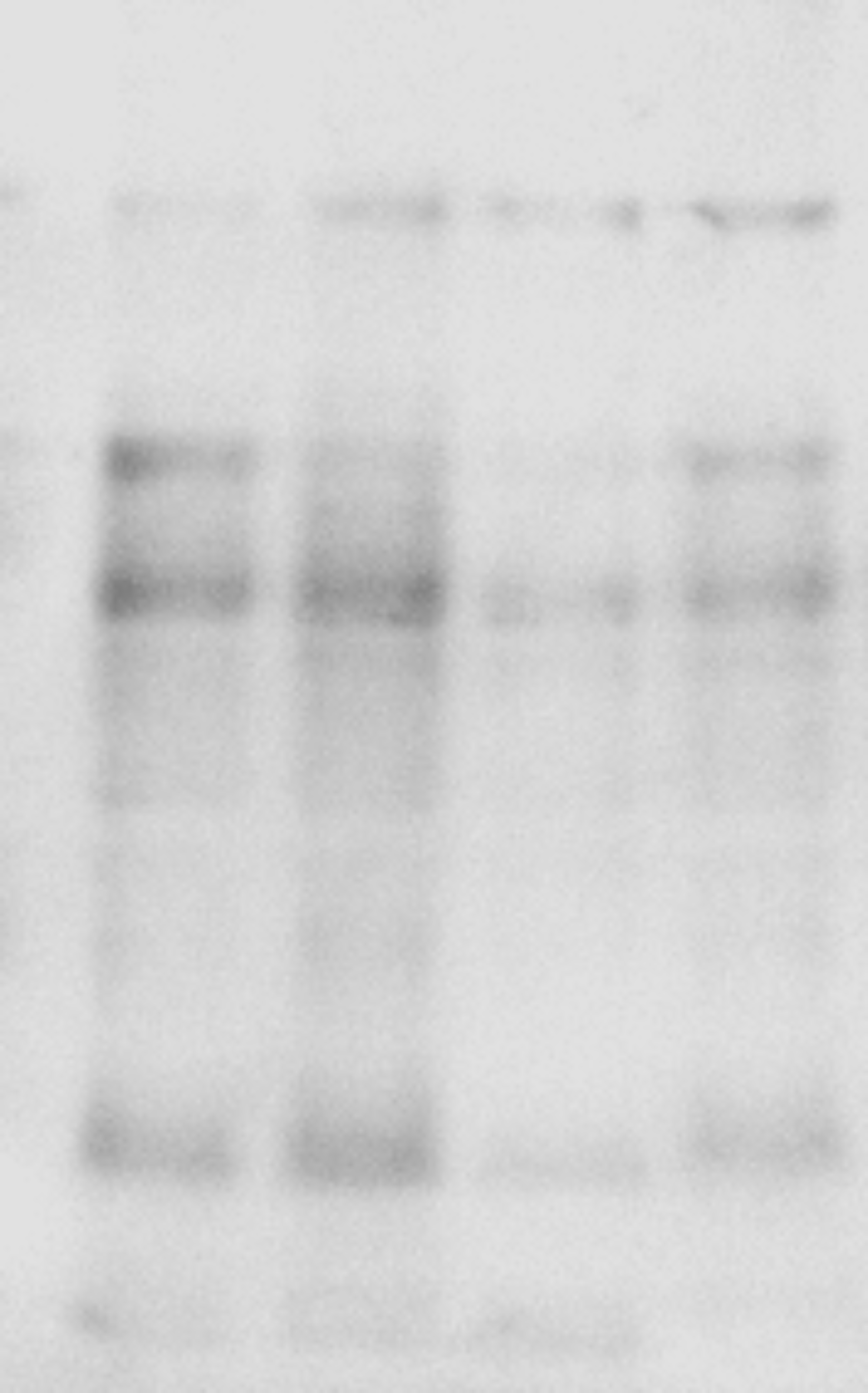

Supplement: Figure 6—source data 3. [file elife-92707-fig6-data3.zip › Figure 6 - Source Data - Unlabelled Western Blots/FIG6A_2.tif]

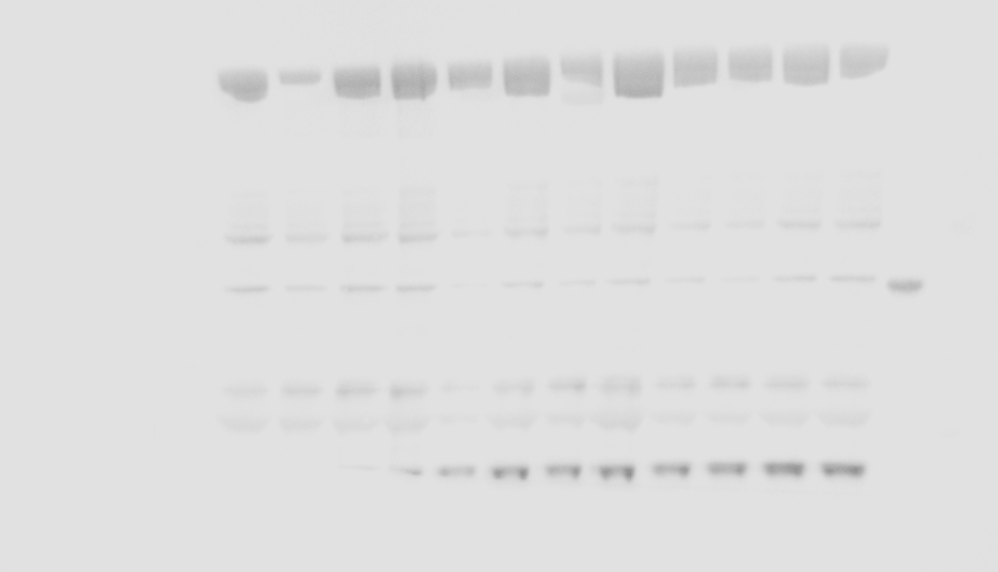

Supplement: Figure 6—source data 3. [file elife-92707-fig6-data3.zip › Figure 6 - Source Data - Unlabelled Western Blots/FIG6F_1.png]

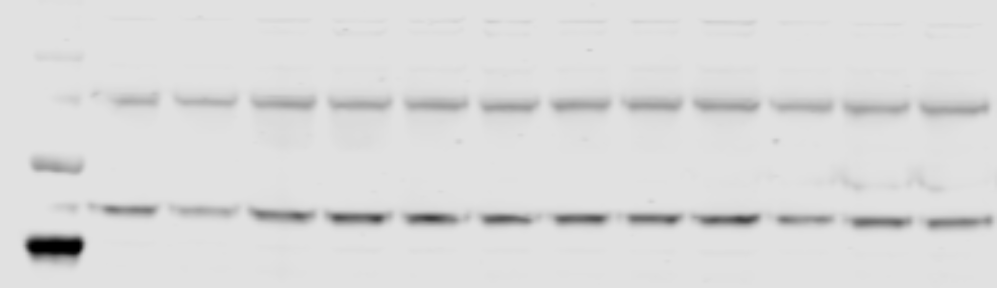

Supplement: Figure 6—source data 3. [file elife-92707-fig6-data3.zip › Figure 6 - Source Data - Unlabelled Western Blots/FIG6D_1.png]

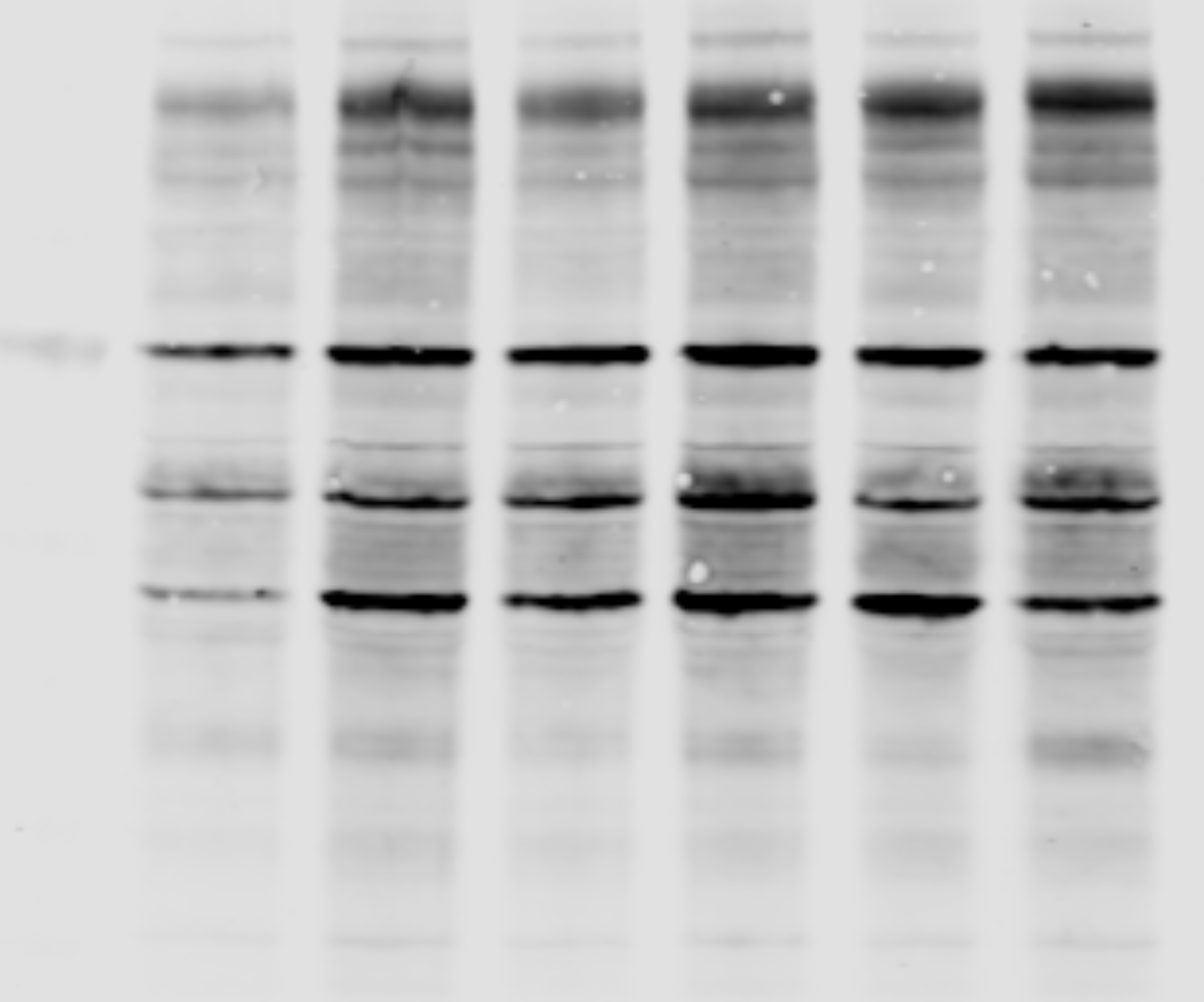

Supplement: Figure 6—source data 3. [file elife-92707-fig6-data3.zip › Figure 6 - Source Data - Unlabelled Western Blots/FIG6A_1.tif]

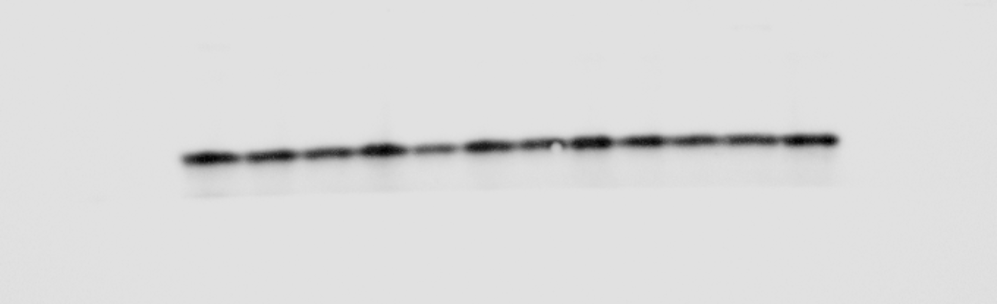

Supplement: Figure 6—source data 3. [file elife-92707-fig6-data3.zip › Figure 6 - Source Data - Unlabelled Western Blots/FIG6F_3.png]

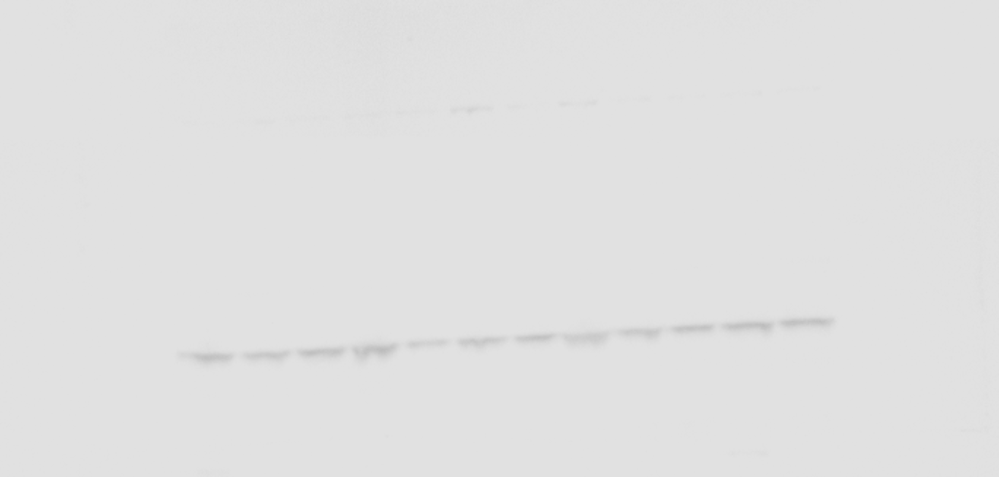

Supplement: Figure 6—source data 3. [file elife-92707-fig6-data3.zip › Figure 6 - Source Data - Unlabelled Western Blots/FIG6F_2.png]

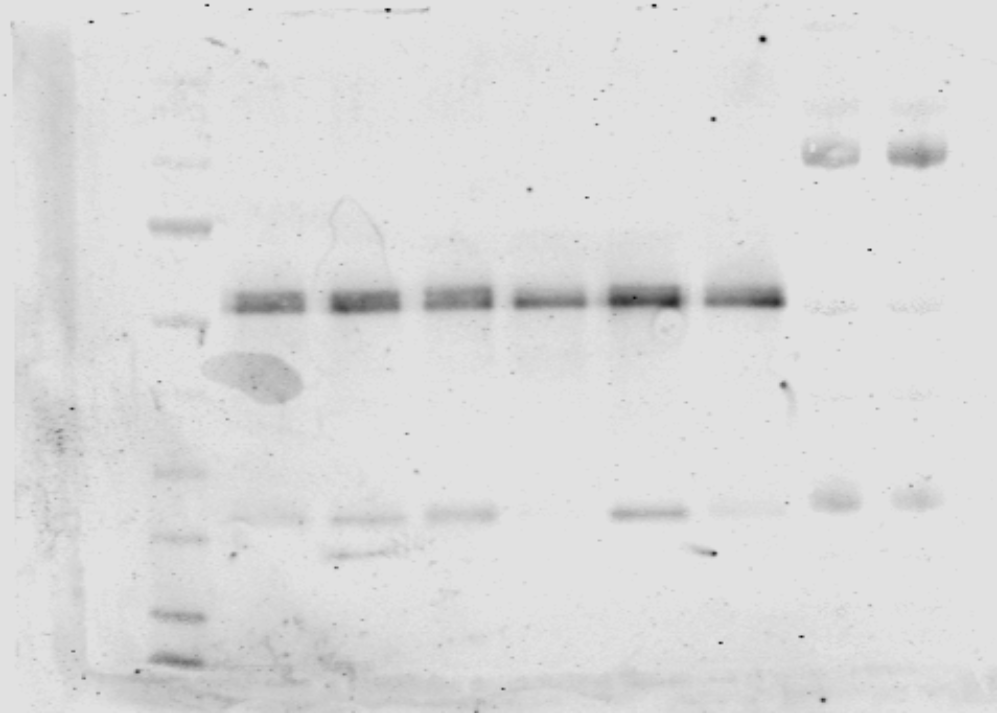

Supplement: Figure 6—source data 3. [file elife-92707-fig6-data3.zip › Figure 6 - Source Data - Unlabelled Western Blots/FIG6B_2.png]

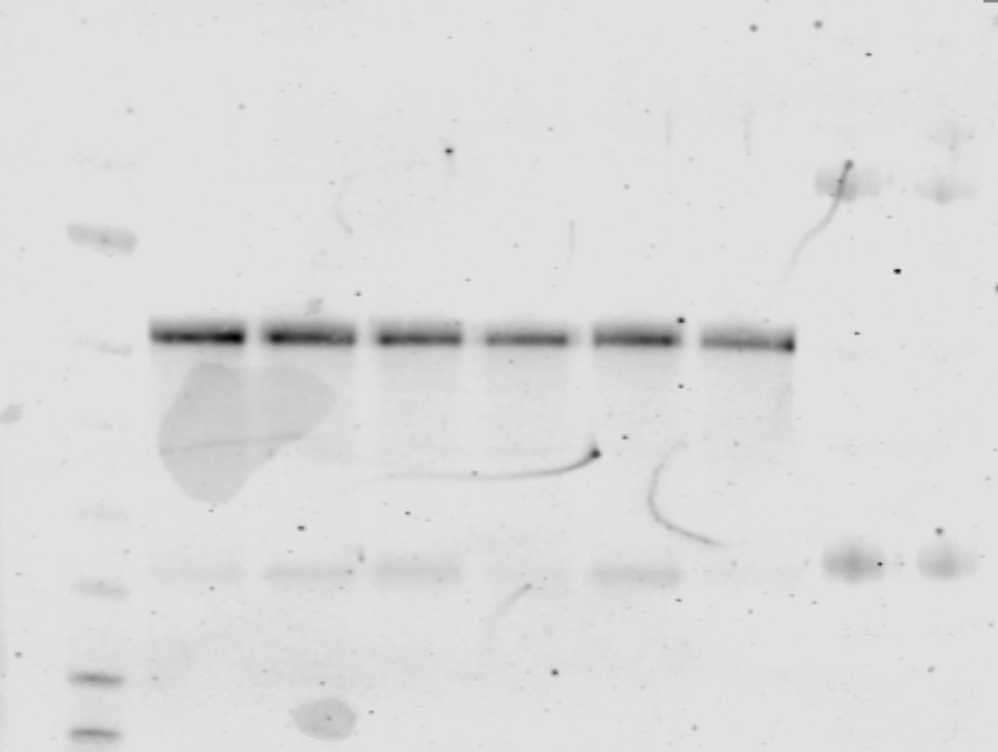

Supplement: Figure 6—source data 3. [file elife-92707-fig6-data3.zip › Figure 6 - Source Data - Unlabelled Western Blots/FIG6B_3.png]

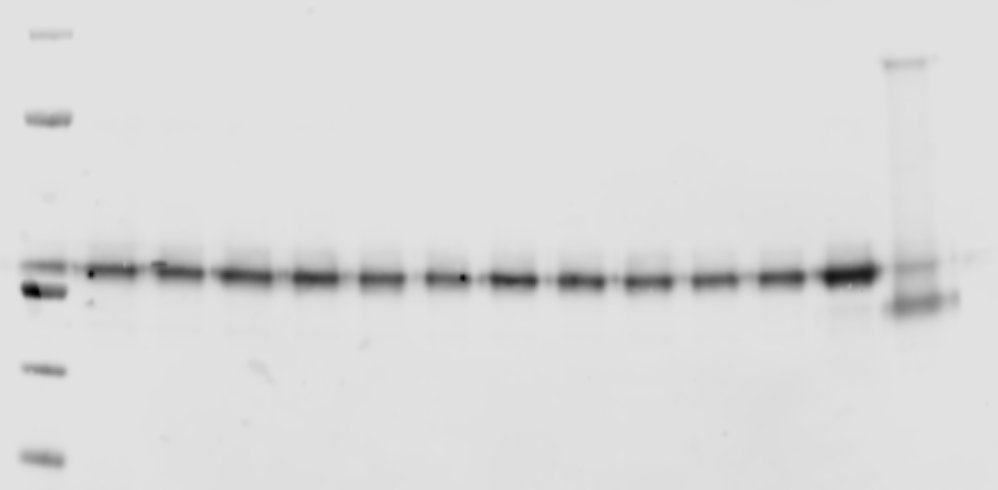

Supplement: Figure 6—source data 3. [file elife-92707-fig6-data3.zip › Figure 6 - Source Data - Unlabelled Western Blots/FIG6B_1.png]

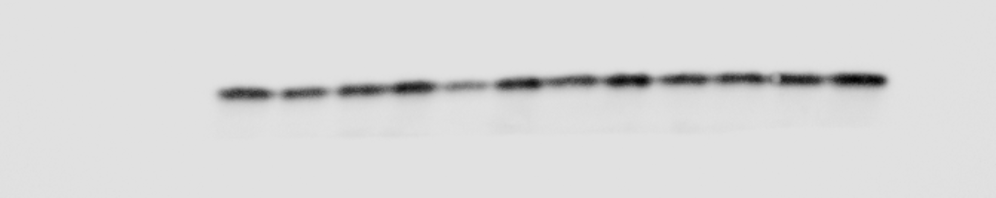

Supplement: Figure 6—source data 3. [file elife-92707-fig6-data3.zip › Figure 6 - Source Data - Unlabelled Western Blots/FIG6F_4.png]

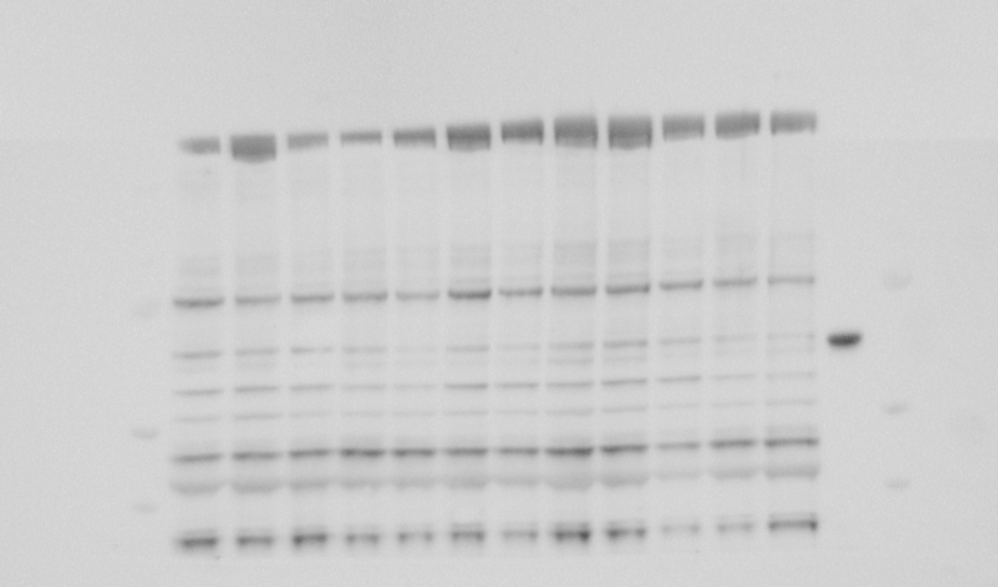

Supplement: Figure 6—source data 3. [file elife-92707-fig6-data3.zip › Figure 6 - Source Data - Unlabelled Western Blots/FIG6E_1.png]

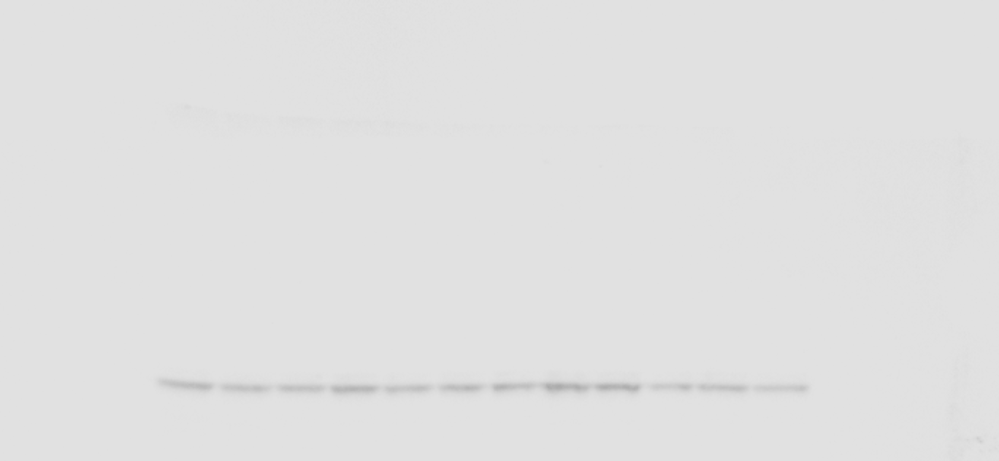

Supplement: Figure 6—source data 3. [file elife-92707-fig6-data3.zip › Figure 6 - Source Data - Unlabelled Western Blots/FIG6E_2.png]

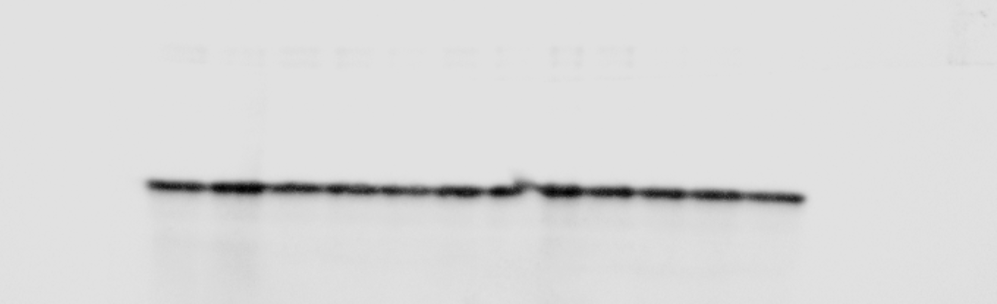

Supplement: Figure 6—source data 3. [file elife-92707-fig6-data3.zip › Figure 6 - Source Data - Unlabelled Western Blots/FIG6E_3.png]

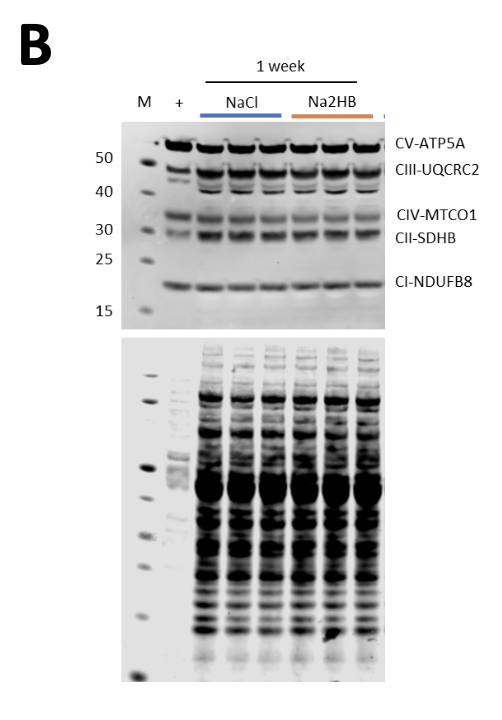

Supplement: Figure 6—figure supplement 1—source data 2. [file elife-92707-fig6-figsupp1-data2.zip › Figure 6 - figure supplement 1 - Source Data - Labelled Western Blots/Figure 6 - supplement 1 - B.png]

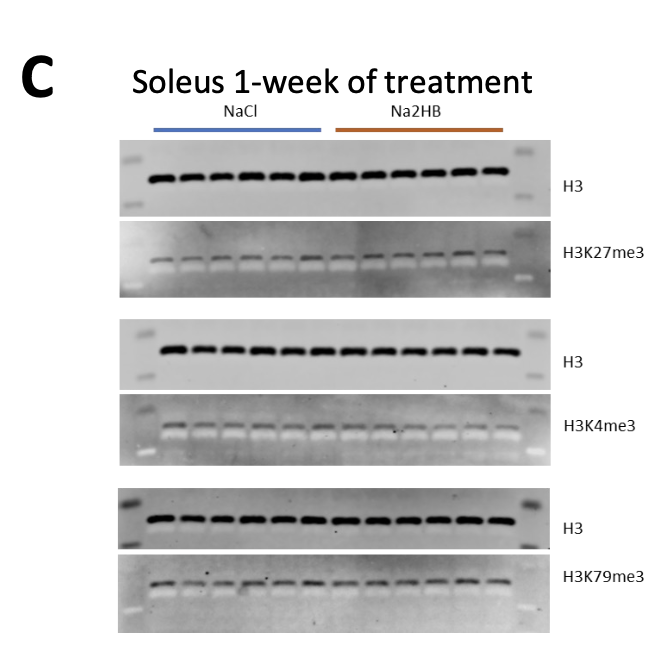

Supplement: Figure 6—figure supplement 1—source data 2. [file elife-92707-fig6-figsupp1-data2.zip › Figure 6 - figure supplement 1 - Source Data - Labelled Western Blots/Figure 6 - supplement 1 - C.png]

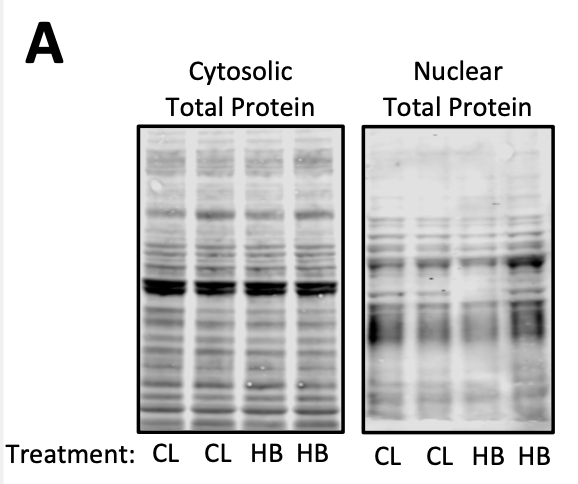

Supplement: Figure 6—figure supplement 1—source data 2. [file elife-92707-fig6-figsupp1-data2.zip › Figure 6 - figure supplement 1 - Source Data - Labelled Western Blots/Figure 6 - supplement 1 - A.png]

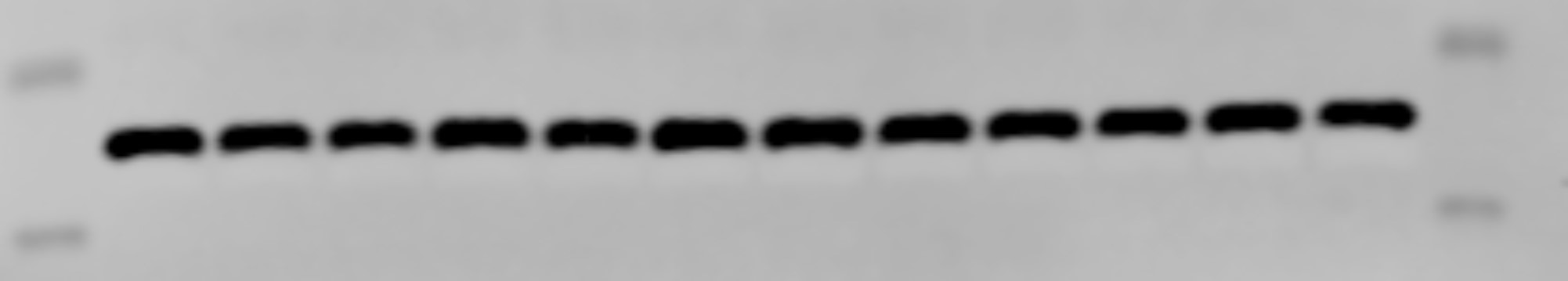

Supplement: Figure 6—figure supplement 1—source data 3. [file elife-92707-fig6-figsupp1-data3.zip › Figure 6 - figure supplement 1 - Source Data - Unlabelled Western Blots/Fig6_figsuppl_1C_H3 for H3K27me3.jpg]

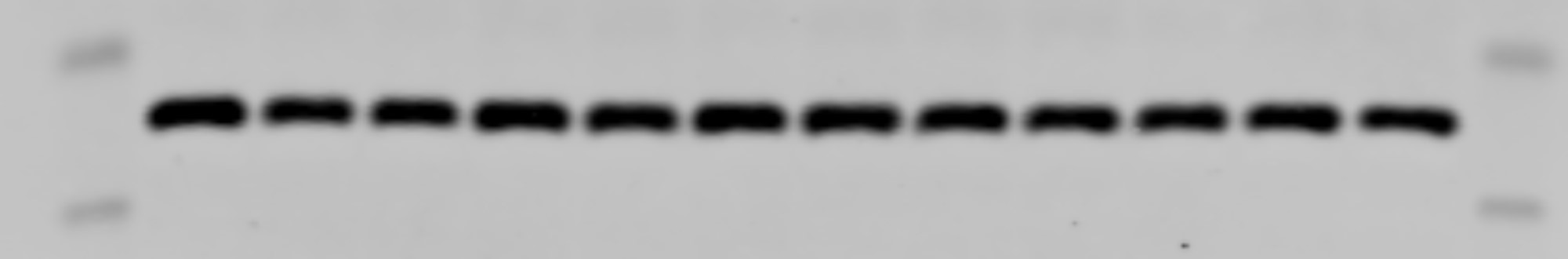

Supplement: Figure 6—figure supplement 1—source data 3. [file elife-92707-fig6-figsupp1-data3.zip › Figure 6 - figure supplement 1 - Source Data - Unlabelled Western Blots/Fig6_figsuppl_1C_H3 for H3K4me3.jpg]

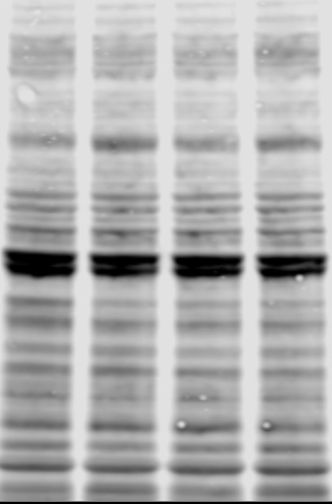

Supplement: Figure 6—figure supplement 1—source data 3. [file elife-92707-fig6-figsupp1-data3.zip › Figure 6 - figure supplement 1 - Source Data - Unlabelled Western Blots/Figure 6_ supplemental 1_A_CytosolicTPS.png]

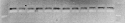

Supplement: Figure 6—figure supplement 1—source data 3. [file elife-92707-fig6-figsupp1-data3.zip › Figure 6 - figure supplement 1 - Source Data - Unlabelled Western Blots/Fig6_figsuppl_1C_H3K79me3.jpg]

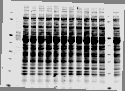

Supplement: Figure 6—figure supplement 1—source data 3. [file elife-92707-fig6-figsupp1-data3.zip › Figure 6 - figure supplement 1 - Source Data - Unlabelled Western Blots/Fig6_figsuppl_1B_TPS.jpg]

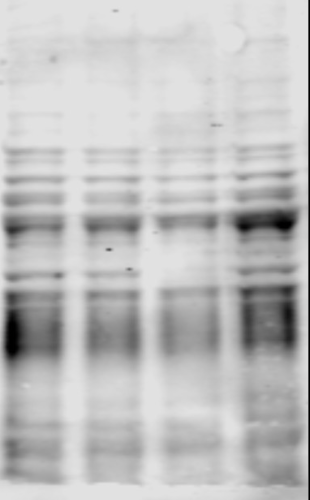

Supplement: Figure 6—figure supplement 1—source data 3. [file elife-92707-fig6-figsupp1-data3.zip › Figure 6 - figure supplement 1 - Source Data - Unlabelled Western Blots/Figure 6_ supplemental 1_A_NuclearTPS.png]

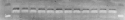

Supplement: Figure 6—figure supplement 1—source data 3. [file elife-92707-fig6-figsupp1-data3.zip › Figure 6 - figure supplement 1 - Source Data - Unlabelled Western Blots/Fig6_figsuppl_1C_H3K4me3.jpg]

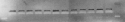

Supplement: Figure 6—figure supplement 1—source data 3. [file elife-92707-fig6-figsupp1-data3.zip › Figure 6 - figure supplement 1 - Source Data - Unlabelled Western Blots/Fig6_figsuppl_1C_H3K27me3.jpg]

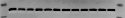

Supplement: Figure 6—figure supplement 1—source data 3. [file elife-92707-fig6-figsupp1-data3.zip › Figure 6 - figure supplement 1 - Source Data - Unlabelled Western Blots/Fig6_figsuppl_1C_H3 for H3K79me3.jpg]

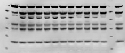

Supplement: Figure 6—figure supplement 1—source data 3. [file elife-92707-fig6-figsupp1-data3.zip › Figure 6 - figure supplement 1 - Source Data - Unlabelled Western Blots/Fig6_figsuppl_1B_OXPHOS.jpg]
