# Supplementary material for: A 2-hydroxybutyrate-mediated feedback loop regulates muscular fatigue
Source: eLife. 2024 Sep 3;12:RP92707. doi: 10.7554/eLife.92707 (PMC11371357; doi:10.7554/eLife.92707)

Figure 6A – Soleus lysates stained for total ADPr

Cytoplasmic extract

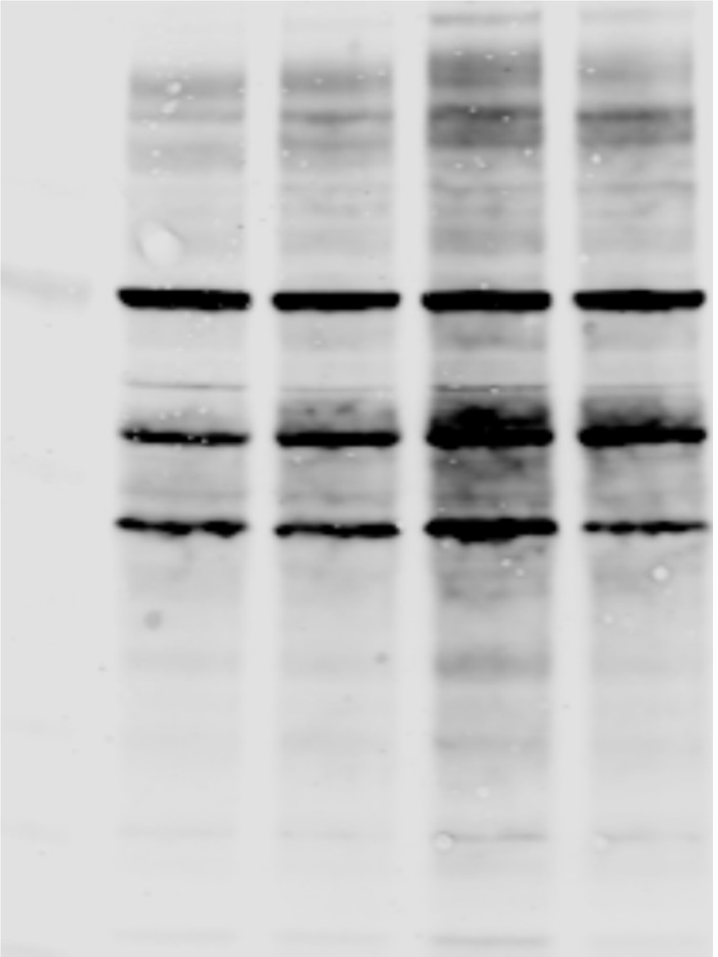

CL CL HB HB

Nuclear extract

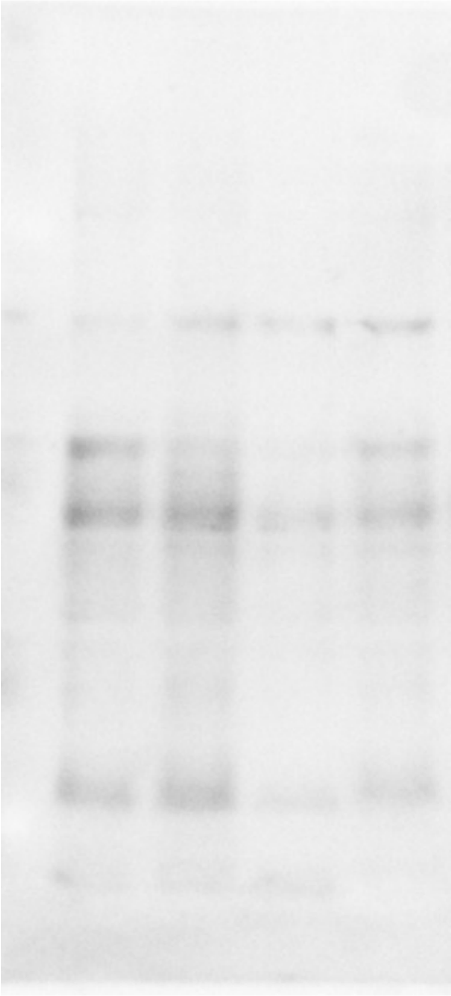

CL CL HB HB

Figure 6B – Soleus lysates IP experiments,

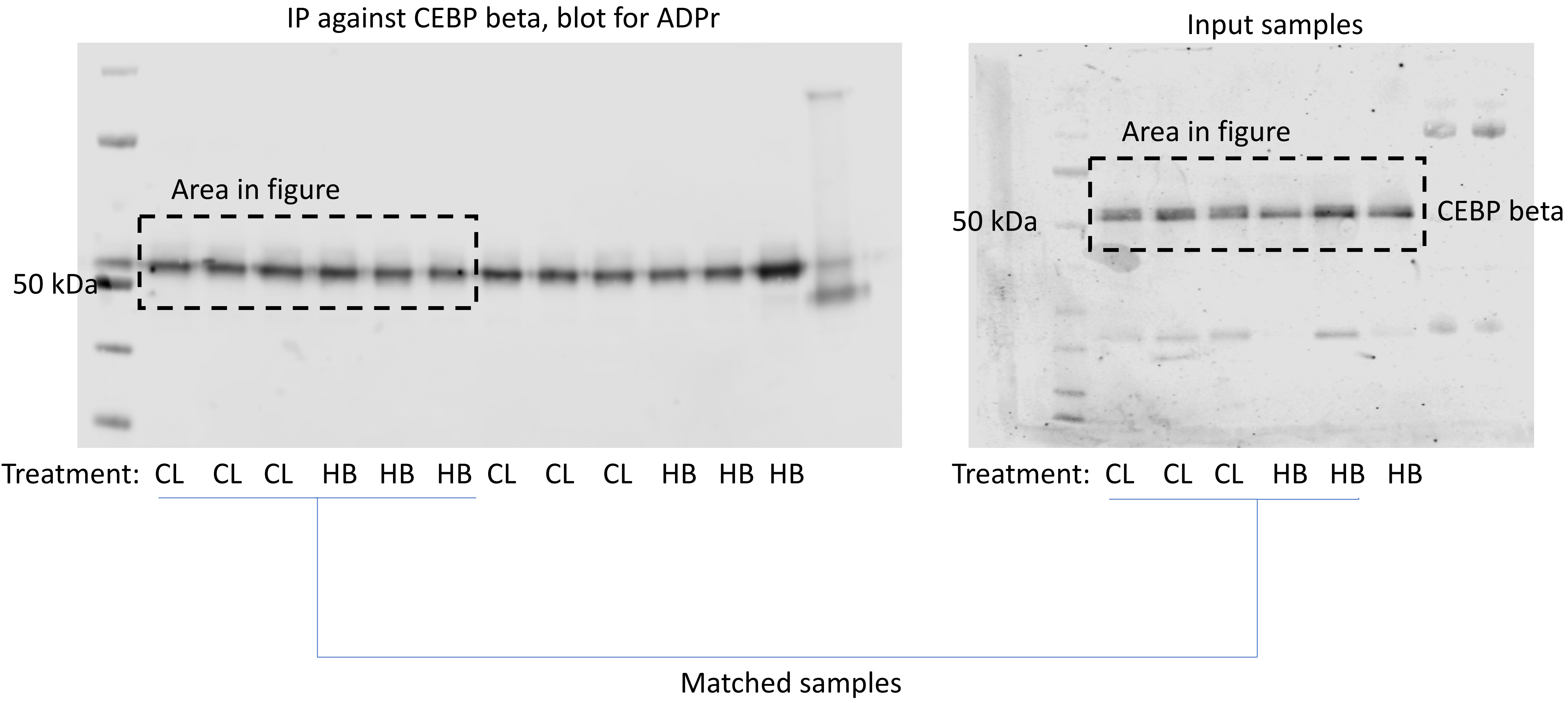

Figure 6B continued – Soleus lysates IP experiments,

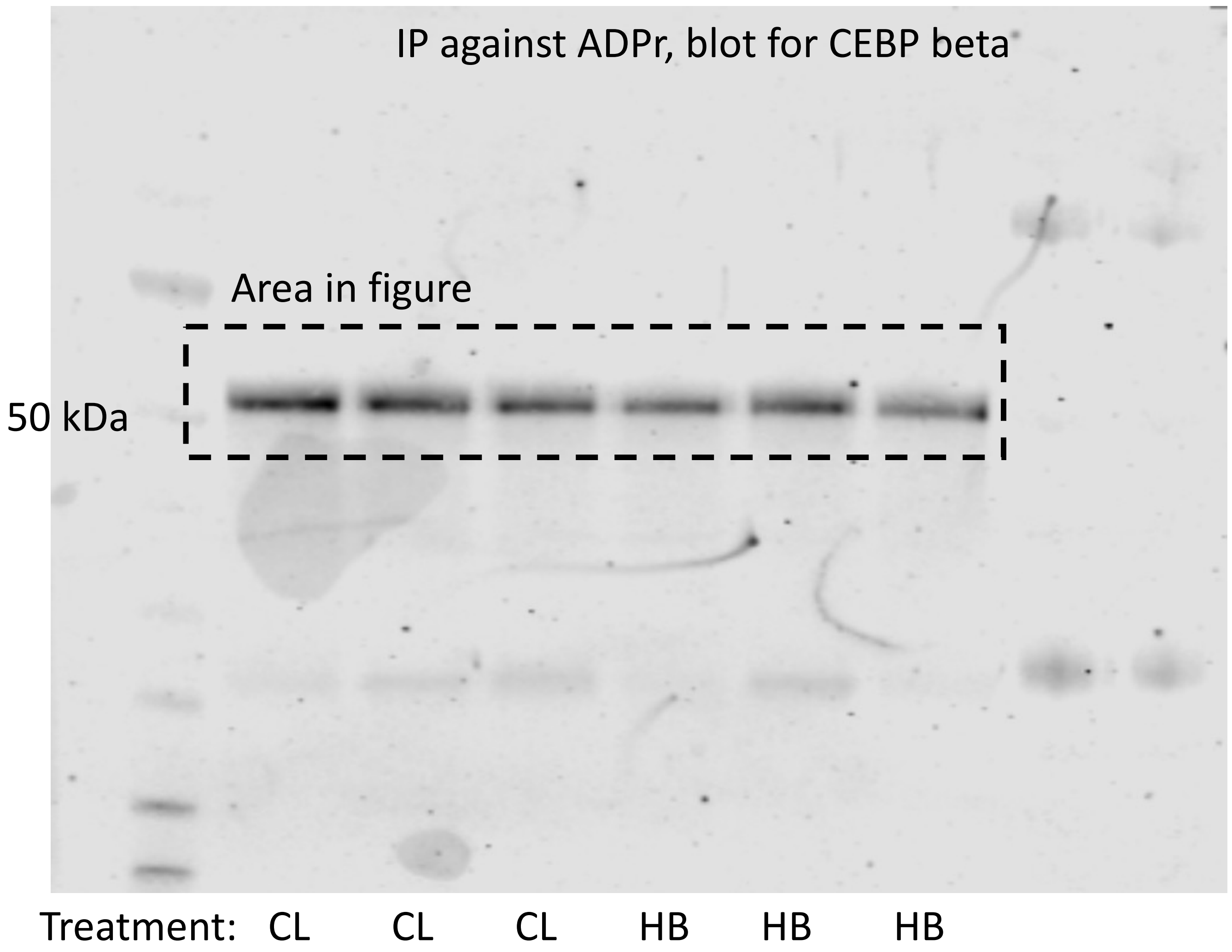

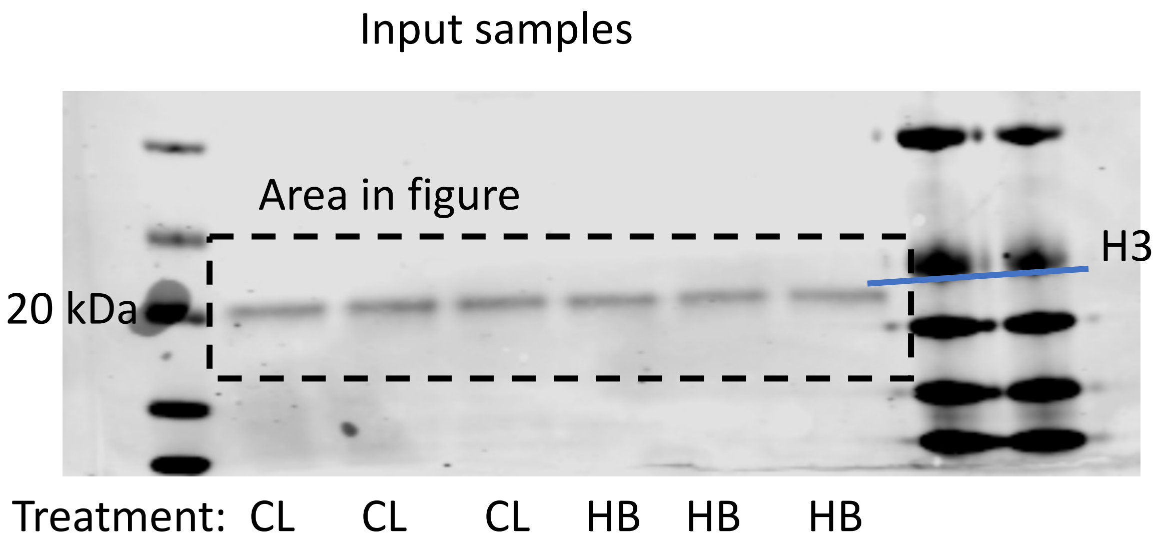

Figure 6D – Soleus lysates

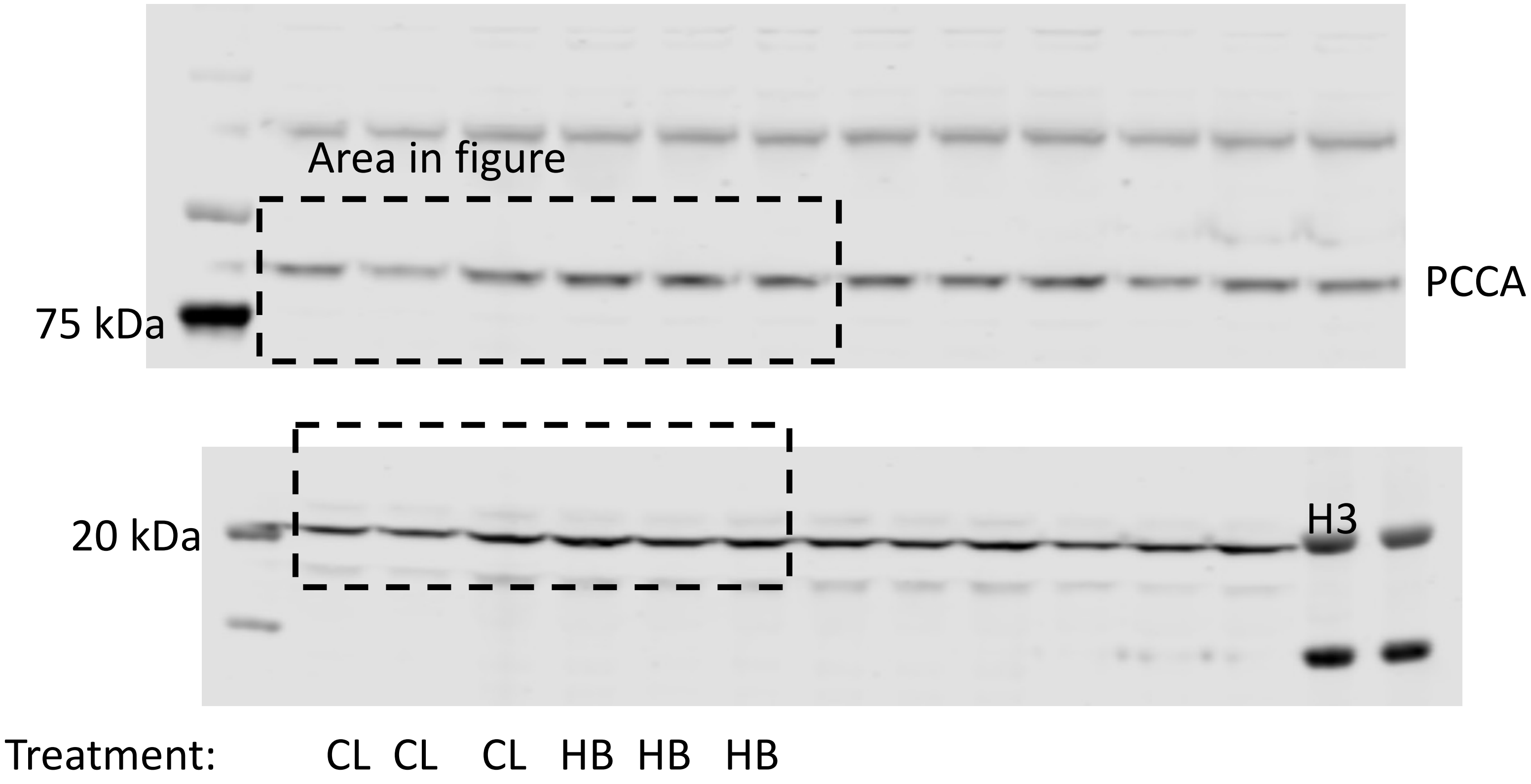

Figure 6E – Soleus lysates

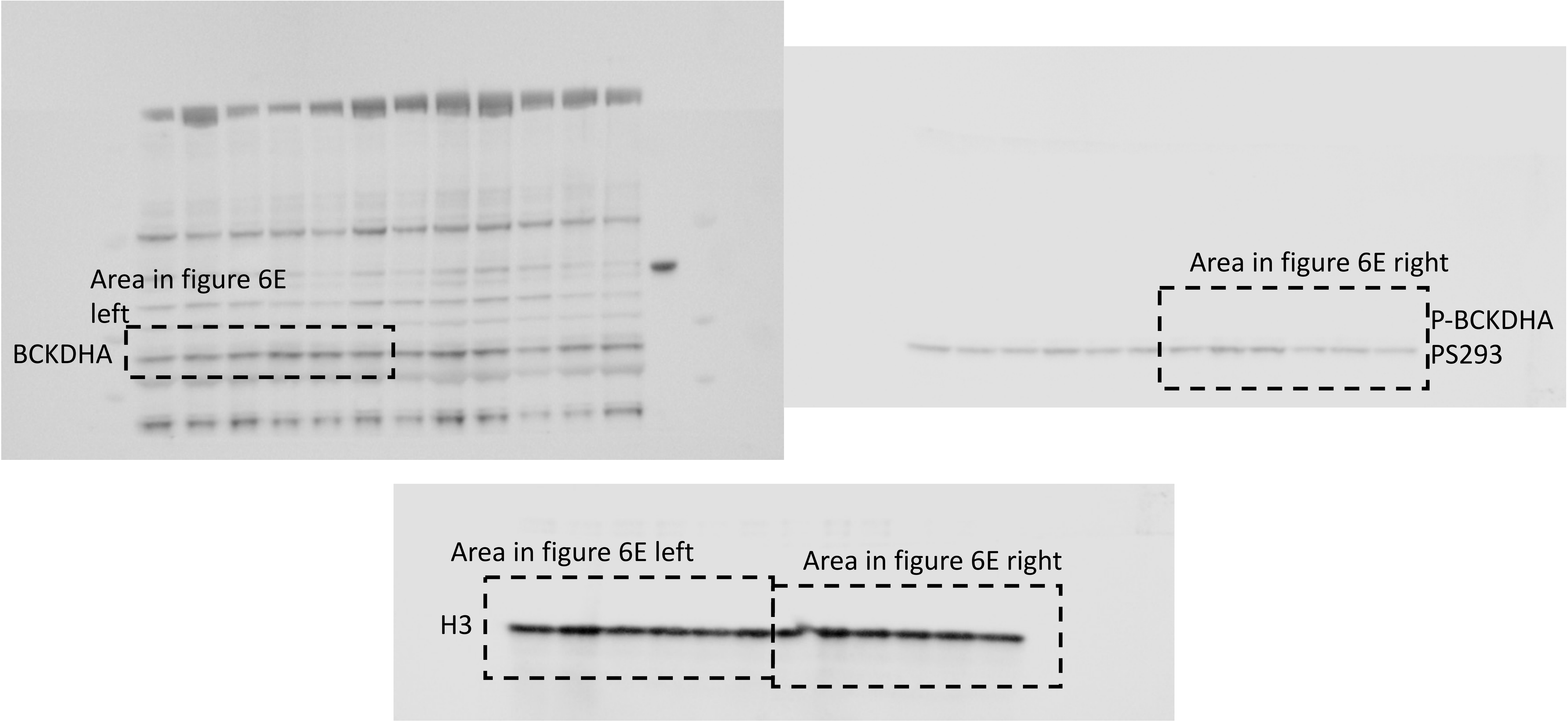

Figure 6F – EDL lysates

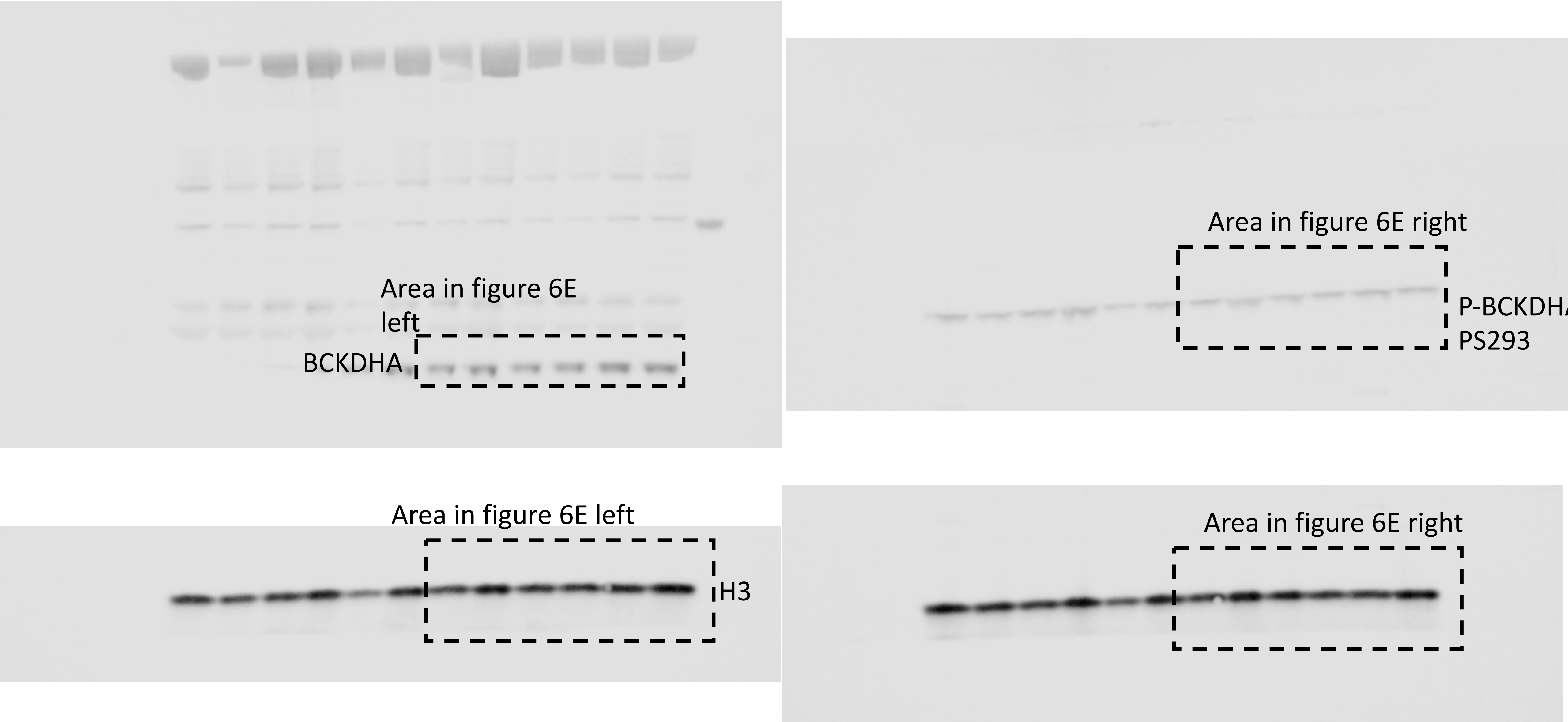

Supplement: Figure 6—source data 2. [file elife-92707-fig6-data2.zip › ANNOTATED WESTERN BLOTS_FIG6.pdf]
