# Supplementary material for: A 2-hydroxybutyrate-mediated feedback loop regulates muscular fatigue
Source: eLife. 2024 Sep 3;12:RP92707. doi: 10.7554/eLife.92707 (PMC11371357; doi:10.7554/eLife.92707)
Supplement: Supplementary file 1. [file elife-92707-supp1.docx]

2HB and 2KB IC50 for isolated PHD and KDM enzymes

|  | 2-Hydroxybutyrate | | 2-Ketobutyrate | |
| --- | --- | --- | --- | --- |
| Enzyme | IC50 (μM) | Inhibition (%) | IC50 (μM) | Inhibition (%) |
| HIFP4H1 | >10000 | <5% | >10000 | <10% |
| HIFP4H2 | >10000 | <5% | >10000 | <5% |
| KDM6A | >10000 | <20% | >10000 | <30% |
| *Values are derived from 3 independent assays.* | | | | |
